# Uncovering individualised treatment effects for educational trials

**DOI:** 10.1038/s41598-024-73714-z

**Published:** 2024-09-30

**Authors:** ZhiMin Xiao, Oliver Hauser, Charlie Kirkwood, Daniel Z. Li, Tamsin Ford, Steve Higgins

**Affiliations:** 1 School of Health and Social Care, University of Essex, Colchester, CO4 3SQ UK; 2 Department of Economics, University of Exeter, Exeter, EX4 4PU UK; 3 Department of Mathematics, University of Exeter, Exeter, EX4 4QF UK; 4 Durham University Business School, Durham, DH1 3LB UK; 5 Department of Psychiatry, University of Cambridge, Cambridge, CB2 0AH UK; 6 School of Education, Durham University, Durham, DH1 1TA UK; 7 Institute for Data Science and Artificial Intelligence, University of Exeter, Exeter, EX4 4QF UK

**Keywords:** Causal inference, Data science, Evaluation, Free school meal pupils, RCT, Subgroup analysis, Randomized controlled trials, Human behaviour

## Abstract

Large-scale Randomised Controlled Trials (RCTs) are widely regarded as “the gold standard” for testing the causal effects of school-based interventions. RCTs typically present the statistical significance of the average treatment effect (ATE), which captures the effect an intervention has had *on average* for a given *population*. However, key decisions in child health and education are often about *individuals* who may be very different from those averages. One way to identify heterogeneous treatment effects across different individuals, not captured by the ATE, is to conduct subgroup analyses. For example, free school meal (FSM) pupils as required for projects funded by the Education Endowment Foundation (EEF) in England. These subgroup analyses, as we demonstrate in 48 EEF-funded RCTs involving over 200,000 students, are usually not standardised across studies and offer flexible degrees of freedom to researchers, potentially leading to mixed, if not misleading, results. Here, we develop and deploy an alternative to ATE and subgroup analysis, a machine-learning and regression-based framework to predict individualised treatment effects (ITEs). ITEs could show where an intervention worked, for which individuals, and to what extent. Our findings have implications for decision-makers in fields like education, healthcare, law, and clinical practices concerning children and adolescents.

## Introduction

Decision makers in healthcare and education often rely on Randomised controlled trials (RCTs) to test the causal effects of psychosocial and educational interventions^1–6^. While the use of RCTs in public policy, healthcare, and education has generally been advocated for by academics with some critiques, such as Deaton and Cartwright^7^ and Biesta^8^, the specific techniques to evaluate RCTs have received less attention. Indeed, only a fraction (less than 1%) of Education Endowment Foundation’s (EEF) £200 million budget has been allocated to the understanding and advancement of evaluation methods. Typically, RCTs are evaluated—and ultimately judged—by the statistical significance of the Average Treatment Effect (ATE) that they achieve for participants in the treatment arm. Interventions that do not have a statistically significant ATE are often discarded as *not meaningful* and, as a result, usually not implemented more widely.

However, while some interventions may not have an effect on average, they might still have a meaningful effect on some individuals for whom the treatment is beneficial. In addition, key decisions in fields like education, healthcare, law, and clinical practices are often about individuals who are not hypothetical averages, which means the evidence that supports the decisions can be inappropriate or even harmful. Indeed, even trials that produce positive and statistically significant ATEs that are further supported by meta-analyses can have unintended consequences for some individuals, as evidenced in recent trials on statin side effects^9^ in medicine. In the largest trial conducted so far to evaluate the effects of school-based mindfulness training on risk of depression and wellbeing in early adolescence, the overall effects were found to be detrimental to adolescents in need of mental health support, despite no ATE and a subgroup of young people who improved their mindfulness and executive skills after the intervention demonstrating improved mental health outcomes^10^. These problems are compounded by the fact that ATEs from large-scale and rigorously conducted and evaluated RCTs often produce small effect sizes sitting within wide confidence intervals^11^, evidence that is hardly actionable for decision makers. This also suggests, ATEs can be positive, even when most participants did not benefit^12^. Therefore, understanding and identifying for whom an intervention works is critical for policy-makers and society at large^12–14^.

Conventionally, researchers rely on subgroup analyses to identify individuals who benefit in trials. Subgroup analyses, in its various forms, usually come with several drawbacks. For instance, choosing which subgroups to analyse, and how to conduct these analyses, which affords multiple degrees of freedom, and risks researchers selectively reporting results that are supportive of their overall conclusion^15,16^. This is essentially “statistical malpractice”^17^. If this occurs, findings may be published that are not reproducible by other researchers, or they may not hold up in direct replication studies, which can shake and erode public trust in science^18^. As such, subgroup analyses are as controversial as they are important: researchers “are damned if they do, and damned if they don’t”^17^ include subgroup analyses in their research.

The EEF, as part of their funding scheme, requires that researchers conduct a subgroup analysis of the treatment for the socioeconomically disadvantaged group of Free School Meal (FSM) pupils. FSM status is one of the most frequently used variables to approximate socioeconomic status in the UK^19,20^. This reporting requirement by the EEF enables us to study current practices in subgroup analyses since it is not specified (or generally agreed upon) how such analyses ought to be conceived and conducted. Previous researchers have cautioned against potential overinterpretation of effects for subgroups like FSM students^21–24^. Although some challenges of subgroup analyses, such as lack of statistical power and unreliable estimation, can be partially alleviated by standardised reporting^21–24^, there are insufficient statistical details in the guidance of key organisations on how to exactly estimate and interpret subgroup effects^25^.

Building on recent insights from data science and machine learning, we propose an alternative to ATEs and subgroup analyses in conventional large-scale RCTs in education, which are an excellent area of research, because policy-makers are keen to improve educational practices and ultimately, equity in pupil attainment and wellbeing and, school-based RCTs, unlike many other policy areas, have become increasingly popular^26–29^. Our approach combines the pragmatism of subgroup analyses, by allowing researchers to learn what works for whom, with the security of relying on a robust and replicable methodology. On the one hand, it is flexible and adaptable to a large breadth of covariates in any given RCT, allowing researchers to study individualised treatment effects (ITEs) from “bottom-up”^30,31^, thus avoiding false positives associated with less rigorous subgroup analyses and unrealistic assumptions often made in the estimation of conventional ATEs. On the other hand, by introducing a principled procedure a priori, it ensures that findings are as robust, reproducible, and transparent as possible. Thanks to recent development in data science and the increase in both quantity and quality of research data, it is easier than before to predict and compare treatment effects at individual and group levels, and increasingly possible to marshal and display telling details on any individual in a given trial, “for the perusal of that individual”^9^, ultimately generating deeper insights into the causal treatment effects of tested interventions^32^.

## Results

### An individualised approach to effect estimation

ATE has long been a quantity of interest in RCTs. However, “the response of the average patient to therapy is not necessarily the response of the patient being treated”^33^. Strong scientific interests in effect heterogeneity do exist, but detecting such variation is not always straightforward in increasingly complex designs. Many EEF trials are, for example, not ideally powered to detect the main effect due to challenges associated with sample size calculations^34^ and resource constraints. As a result, as we will demonstrate later, current methods to identify individuals who benefited from an intervention via subgroup analyses are not always helpful. Unsurprisingly, estimates of effects for FSM students in EEF trials usually come with the caveat, “should be interpreted with caution”.

**Fig. 1:**
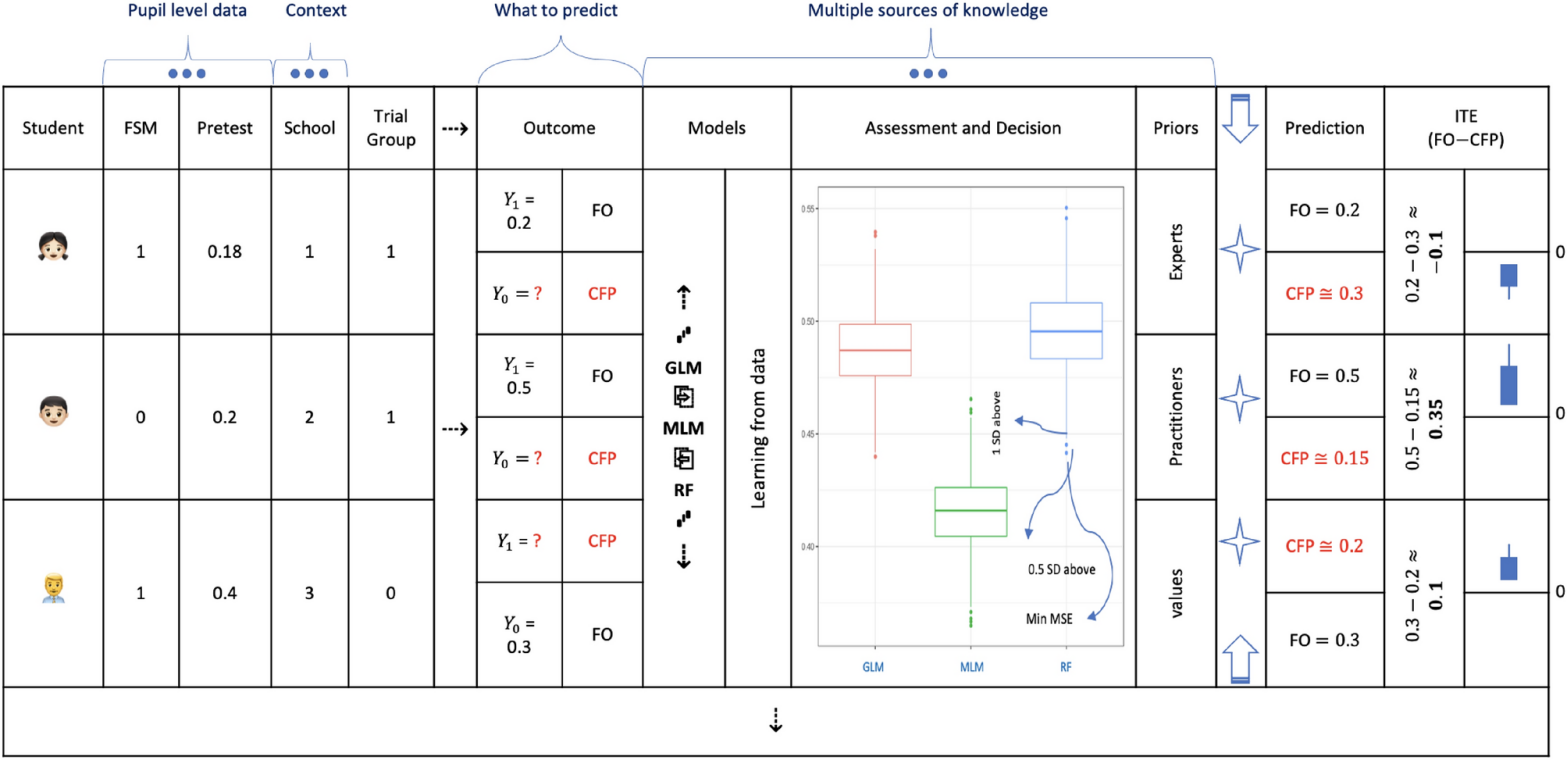
ITEs through a graphical journey. With student- and school-level data, such as FSM status and Pretest for the former, and School ID for the latter, multiple models compete on the same data to predict counterfactual outcomes (CFP) for individual students. FO is the factual outcome observed under the treatment condition a student actually received in an intervention. The difference between FO and CFP is used to re-train the best performing model for the re-prediction of ITEs, which are then visualised as bar plots for unique individuals. Note that the generation process for ITEs is iterative and the arrows pointing to four directions represent the hidden procedures. There are also many more variables and even more observations, which are visualised as dots and dotted arrows.

The alternative approach we propose here taps into the advancement of predictive algorithms in machine learning^35–38^. It focuses on the differences between two potential outcomes^38–40^, often an observed factual and an unobservable counterfactual outcome^41,42^. We call the differences individualised treatment effects for a given individual in a trial that has taken place (see Fig. 1 for an illustration).

The evidence generated from the individualised approach is thus unique to specific individuals according to their observed characteristics. For each individual in an educational RCT that has taken place for instance, we observe the intervention arm assigned $T \in \mathcal {T}$, the intervention outcome $Y\in \mathbb {R}$, and the student’s pre-intervention and school-level characteristics, all captured by an *m*-dimensional vector $\textbf{X} \in \mathcal {X}$. (In line with published works^33,38,40,43^, we use upper- and lower-case letters to denote random variables and their realised values respectively, and bold letters to represent vectors in the data.) For simplicity, we let $T = 1$ denote the treatment group and $T = 0$ the control or business-as-usual group, hence $T\in \{0,1\}$. Without loss of generality, we assume a higher value of *Y* suggests a better outcome.

The ATE in a conventional RCT evaluation is defined as $\mathbb {E}(Y|T=1)-\mathbb {E}(Y|T=0)$. When calculating ITE, we have an observed factual outcome, and need to predict a *counterfactual* outcome, had the student been assigned to an alternative intervention arm. That is to say, for a student with covariates $\textbf{x}$ in the treatment group $T=1$, we observe the factual treatment outcome, denoted as $y_1(\textbf{x})$. We utilise the observed data to predict the unobservable counterfactual outcome, denoted as $\hat{y}_0(\textbf{x})$, and calculate ITEs as $y_1(\textbf{x})-\hat{y}_0(\textbf{x})$.

A number of models can be deployed to predict potential outcomes. Given a pre-specified model $f(\textbf{x};T)$ and the observed covariates $\textbf{x}$, the true data generation process for the student *i* is

$$\begin{aligned} Y_{i} = \underbrace{ f(\textbf{X}_i, T_i) + \xi _i(\textbf{X}_i, \textbf{U}_i, T_i) }_{\mathbb {E} \left[ Y_i | \textbf{X}_i, \textbf{U}_i, T_i \right] } + \varepsilon _i, \end{aligned}$$

where the first two terms represent the true conditional expectation, and the last term is the irreducible error around it^33^. Unfortunately, the true data generation process is unknown, even in the absence of $\varepsilon _i$, as *f* will always differ from the true process by $\xi _i(\textbf{X}_i, \textbf{U}_i, T_i)$, where $\textbf{U}_i \in \mathcal {U}$ represents *unobserved* and *unobservable* covariates, such as culture, tradition, and value-based decisions. That is, we cannot gather data on all possible covariates in a study, and we also need to recognise that researchers bring different assumptions into the data generation process at collection, pre-processing, and analysis stages^44^. Yet, given sufficient covariates, these models *can* yield insightful information about ITEs.

As an illustration of the individualised approach, we focus on one EFF-funded dataset from a trial called Chess in Schools^45^. This project had 100 schools randomly assigned to either intervention or control, involving 4009 pupils. Intervention schools taught children how to play chess over a year, whereas control schools were business-as-usual. The primary outcome was Key Stage 2 Maths score—an important standardised test taken by UK pupils usually at age 11—1 year after the intervention.

We highlight the above project for the following reasons. First, it has a relatively large sample size and the highest possible security rating of five padlocks, which indicates high internal and external validity^46^. Second, at a total cost of £689,150, the project represents some intensive efforts to improve maths skills, yet the reported overall effect size of $0.01 (-0.15, 0.16)$^45^ might suggest that it was not a worthwhile investment, prompting the question whether this intervention did have some non-negligible and educationally meaningful effects for some students, given the report that 50% of the pupils said “they liked the chess lessons a lot” and “teachers were very positive about the intervention and its impact on pupils’ skills and behaviour”^45^. Finally, the dataset has many observed covariates, which makes it suitable for this individualised approach. Any other RCT that fulfils the above criteria would make an ideal candidate for this approach.

**Fig. 2:**
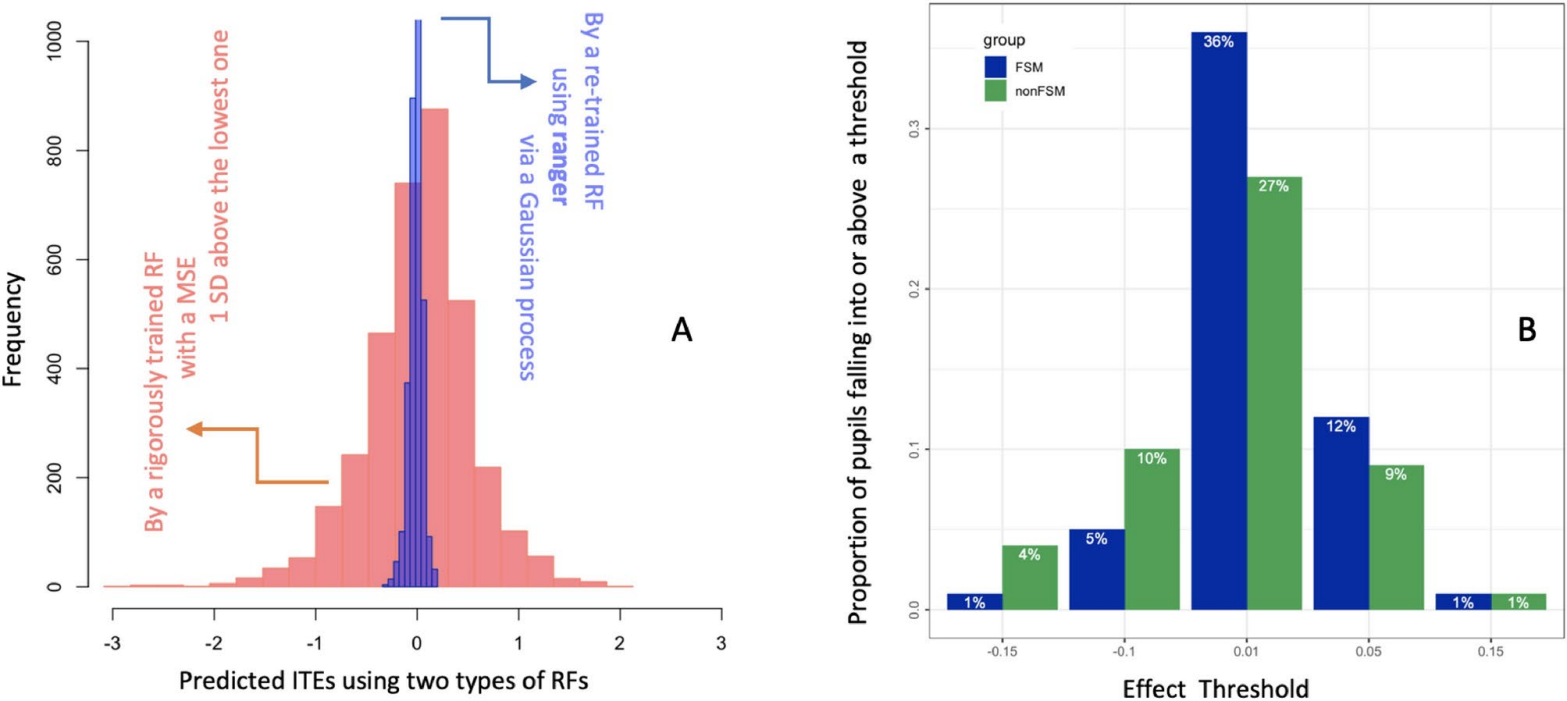
ITEs for use. (**A**) Histograms of two distributions of ITEs from two types of RF, one predicting one potential outcome and the other re-trained to predict the differences of two, i.e., ITEs. (**B**) ITE thresholds used to visualise the proportions of FSM and Non-FSM students predicted to benefit (if positive) or lose (if negative) from the intervention by at least a chosen amount.

Using generalised linear models (GLM), multilevel linear models (MLM), and random forests (RF) as candidate models, we made a chain of predictions for our ultimate quantity of interest, ITEs, of which two distributions are shown in Fig. 2A. For further details about model selection and discussions on the target of prediction, please see the Methods section.

With ITEs from the optimal model, namely, a re-trained RF, we visualised in Fig. 2B the proportions of FSM and Non-FSM students at different ITE thresholds, which were set according to what we knew from the literature and understood about the trial under investigation. For instance, either the overall effect size or that for FSM students reported by the original evaluation team is 0.01^45^, and the average of all the EEF trials funded to date is about 0.05^11^. Other values in the figure reflect the distribution of our predicted ITEs. We found that 36% of the FSM students in the study benefited by at least 0.01 *sd* from the Chess intervention, whereas 27% of the Non-FSM students gained by the same amount. The pattern continues, such that more FSM students benefited from the intervention than Non-FSM students, up to 0.05 *sd*. A small fraction, equally made up of FSM and non-FSM students, benefited by 0.15 *sd*. On the negative side, fewer FSM students were worse off than their Non-FSM counterparts by 0.1 or 0.15 *sd*. Taken together, the individualised approach demonstrated that the intervention was more beneficial to FSM students than to Non-FSM students, amplifying the positive feedback in the form of qualitative data given by the pupils and teachers actually involved in the trial^45^.

While the above statistics are telling of the benefits, it is important to note the uncertainties, either epistemic or aleatoric, surrounding those percentages. The point demonstrated above is not about an *absolute* reality of how many, *precisely*, FSM or Non-FSM students benefited from the intervention by how much, *exactly*. Different models, no matter how well calibrated they are via cross-validation or bootstrapping, could generate different distributions of ITEs that might result in different comparison statistics. The resultant statistics might also differ had we chosen different prior knowledge about the effect thresholds. The point we wanted to demonstrate is that significantly more FSM students than their Non-FSM counterparts benefited from the seemingly ineffective intervention as measured and reported using the official ATE, after we incorporated *some* uncertainties associated with model calibration and prior knowledge informed by the literature to date.

**Fig. 3:**
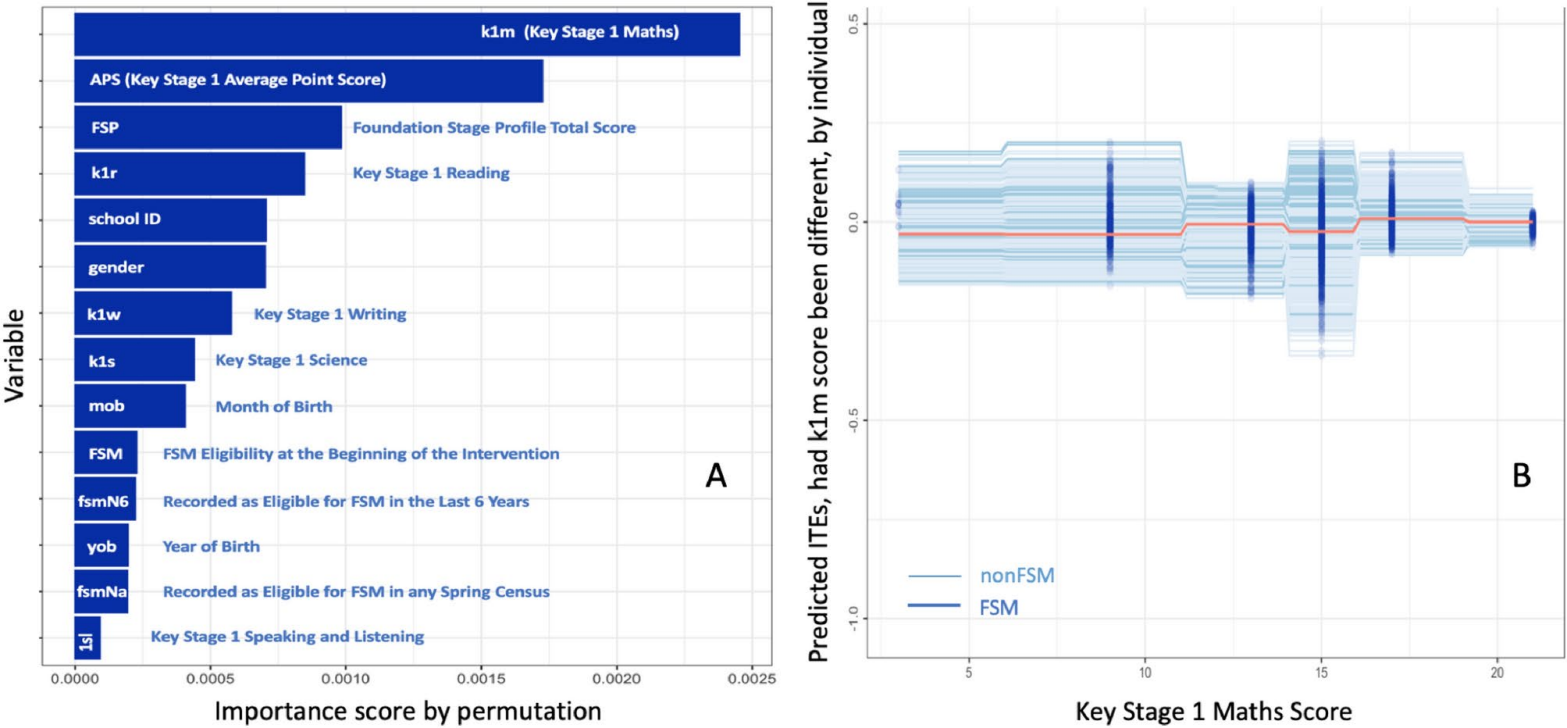
ITEs and non-statistical knowledge. (**A**) The re-trained RF algorithm allows us to see the most important predictor of ITEs, which, in this case, is Key Stage 1 Maths. (**B**) Expected ITEs for different individuals, be they FSM or Non-FSM students, had their performances on Key Stage 1 Maths been different. The orange line represents an average student.

When acting on or interpreting the ITEs, we may incorporate non-statistical knowledge. For example, according to Fig. 3A, baseline measure k1m, or KS1 maths score, is the most important predictor of ITEs, which is followed by APS (KS1 Average Point Score) and FSP (Foundation Stage Profile total score), both extracted from the National Pupil Database. Given this information, educators and decision-makers who know the students and/or their schools best can intervene by focusing on what is practically possible and most important to do and when. However, the variables ranked in the importance plot are observed covariates in the data, it does not mean that no other factors that were not observed in the study are at least as important as k1m.

In other studies, the most important variable observed may not be educationally desirable^8^. Suppose corporal punishment, which is easy to measure, is one of the strongest predictors of ITEs, it does not mean that we should implement that policy. This is exactly when non-statistical knowledge and educational, social, and psychological theories have important roles to play in identifying variables to use for the prediction and helping interpret the ITEs then produced for policy use.

Suppose again decision-makers will listen to us and choose to intervene by investing more in maths education at KS1. They want to see by how much an individual would be (made) better or worse off, had the student’s performance on the subject been different as a result of the investment. And what would the effect of the intervention be like if the student is eligible for FSM?

To answer the questions posed above, we visualised the effects for the individual under different hypothetical scenarios in Fig. 3B by following an individual conditional expectation (ICE) plot procedure, which, in the words of the authors^47^, allowed us to “peek inside the black box” of the RF, and see how ITEs vary with the covariates that characterise each individual. With the assumption that the RF has successfully learned the relationships present in the dataset, we can for each individual vary each covariate one at a time through its full minimum-to-maximum range, and see how ITEs are expected to change for each individual “if they had this character instead”.

Figure 3B shows the expected influence of KS1 maths score on ITEs at an individual level (each individual in the dataset has her/his own line). We can see that, in a general sense, ITEs tend to be *associated with* FSM and k1m. ITEs are higher at higher k1m scores. By also differentiating the lines based on the FSM status of an individual, we can observe an interaction effect between FSM and k1m, and it appears that FSM individuals with k1m scores between 10 and 20 would benefit more from the intervention than their Non-FSM counterparts.

The highlighted words “associated with” imply that it would be ethically impractical to make a student eligible for FSM even it is practically possible to do so. Similar ethical and perhaps legal considerations apply when other covariates such as gender and ethnic backgrounds are involved, although an algorithm might suggest that those covariates would be important to predict ITEs.

Once again, we have shown that the individualised approach is a collaborative endeavour to evidence generation and use and it involves multiple stakeholders in education. To best serve the students we care about most, it invites academics from diverse backgrounds and people with domain knowledge and professional experiences to collaborate and co-produce evidence that is relevant to individuals and groups alike and actionable for policy-makers and practitioners.

### Conventional approaches to subgroup analyses

To justify the need for an alternative approach to effect size estimation (re-)using data from large-scale RCTs, we must show how conventional ways of analysing the data pose substantial challenges. Following an established approach to subgroup analysis in the evaluation of EEF trials, we also employed, as specified in Supplementary Information (SI), an ordinary least squares (OLS) model, which has as covariates, a treatment-FSM interaction term plus pre-test, a baseline measure available in almost all EEF trials. For each outcome, we obtained the sample size used for the interaction test and the *p*-value associated with the interaction term. We then deployed a multilevel linear model (MLM) recommended by the EEF, also documented in SI, to estimate effect sizes within the subgroups of FSM and Non-FSM students using eefAnalytics, an R package specifically developed to estimate effect sizes for RCTs funded by the EEF. The two subgroup effect estimates were then compared with the *p*-values from the interaction tests, which indicate if the differences between the two separately estimated subgroup effect sizes are statistically significant.

In total, we examined 84 outcomes from 48 projects (see Table S1 for details), which are distinct and separate projects designed and evaluated by independent teams in different years. As we had access to the raw data, this analysis differs from a standard meta-analysis. Instead, we reported and calculated, for each outcome (see Figs. S1–S13), three effect size estimates, their associated sample sizes and 95% confidence intervals. The first, for reference only, is the overall effect size every EEF project reported for all the students involved in each trial. The other two are separate estimates produced by us for Non-FSM and FSM students.

The results of our re-analysis show that, only 6 out of the 84 outcomes are statistically significant for FSM students, which means, consistent with the literature on overall effect sizes of EEF studies^11^, conventional regression analyses focusing on ATEs often produce results that are non-actionable, even when participants’ lived experiences as reported in process evaluations show otherwise^45^. One reason, in addition to those given elsewhere^7,11^, this may be the case is that we conducted the same analysis across all studies, thereby holding all analyses to the same standards. Arguably, of course, our analyses following some conventional approaches to subgroup analyses are not necessarily the only “correct” ones. However, this gives further credence to the need for alternatives to impact evaluation and better (re-)use of research data from trials we have invested so much in.

## Discussion

In line with previous research, we proposed an individualised approach here to effect estimation, which employed three types of predictive algorithms to first predict how individuals would respond to different treatment options. We then quantified the differences in potential outcomes at individual level, which were subsequently used to re-train the best of the three candidate models to predict the ultimate quantity of interest, namely, ITEs for unique individuals. These highly individualised effect predictions can be utilised to evaluate an intervention that has taken place, thus making the best use of research data and offering an alternative to conventional ATE and subgroup analyses.

The ITEs can be further examined with other variables, such as FSM status, a variable used for subgroup analyses in EEF trials. While we focus on ITEs, it is still important to see if and how they converge with the ATE when aggregated, as schools and policy-makers in education and healthcare often need to consider participants in groups, be they classes, year groups, schools, or districts. We demonstrated that the results from the individualised approach could reproduce the original ATE *and* answer policy-relevant questions about subgroups of students, such as those from disadvantaged backgrounds who experience worse education.

In sum, we have shown that, while conventional approaches to effect size estimation in policy interventions often resort to aggregated measures of impact, such as ATE or conditional ATE^43,48^, these evaluations take little note of individual characteristics that may alter individual responses to interventions. After all, an intervention that worked well *on average* may not be the best option for all, while a substantial benefit for some might be worth having, even if there was no ATE^49^. Any subgroup analysis, or heterogeneous treatment effects analysis, is one step closer towards a more sensitive estimation of individual responses to an intervention^50^, but current practices are not standardised. Heterogeneous causal effect prediction using causal forests permits *statistical* inferences and quantifies their uncertainties, thanks to its asymptotic properties associated with a normality theory^40,43^. Nevertheless, it is worth pointing out that no method can lead to a truly individualised effect^51,52^: no individual can ever be in both the control and the treatment arms at the same time. However, the methods proposed here and elsewhere by others^38^ can get closer to an ideal individualised effect. Future research will continue to develop consistent and efficient ways to construct these predictions, as the current process is rather “data hungry”, often with half-half sampling split for feature space construction and effect estimation. As Wager & Athey note, however, the sampling split rule is arbitrary and “still in its infancy”^40^.

As in any research, our individualised approach has its own limitations. First, while it focuses on evidence that is actionable, the prediction procedures could have been more dynamic (i.e., each individual may have their own best prediction algorithm, rather than *a* best performing algorithm for all individuals) and the uncertainties surrounding those ITEs are yet to be formalised and refined. Second, we could have simulated some data from an RCT where the intervention effect is zero and no relationships exist amongst covariates. If the predictive algorithms fail to identify individuals who benefit from the “trial”, we would be much more confident about the approach. It would be even better if we can test the approach in multi-phase trials and/or deploy it to predict outcomes in longitudinal studies.

Finally, while our approach has shown to work effectively in education, it is worth emphasising that it can be applicable and relevant in any area of science and policy—from tax collection to medical trials to public health. The importance of individualising treatment effects is particularly critical in areas of rapid development, such as testing of drugs or vaccines in N-of-1 trials^53^, or of policies that encourage certain behaviours for the public good^54^. Take, for example, the COVID-19 crisis: first, vaccine trials might benefit from the individualised approach, in that even a vaccine that only works for a relevant subgroup (e.g., patients with underlying health conditions) would be a much-needed advancement to battle the deadly disease; second, encouraging social distancing may take different forms and policy-makers would be well-advised to understand how different subgroups (e.g., the elderly, young people, and key workers) might respond to different messaging.

## Methods

For the demonstration, the Chess in Schools dataset we constructed has 16 variables, including the Key Stage 2 Maths outcome measure and treatment indicator. Pre-treatment covariates consist of Key Stage 1 measures and pupil-level characteristics such as FSM status. To be consistent with the way most EEF evaluators dealt with missing data in their primary analyses, we also removed all the rows with missing data, which is less problematic to machine learning algorithms such as random forests than it is to inferences based on probability theories^55^. We ended up with a sample of 3514 complete cases, which is unsurprisingly smaller than 3,865 the evaluation team reported and 3695 for the interaction test in Fig. S1, as the variables used to construct the data are different.

To conduct model selection, we first randomly split, via bootstrapping, the observed RCT data into two disjoint training and testing subsets. The training set was used to train candidate models, and the testing set to assess their performances. In each bootstrapped re-sample, an outcome of interest was predicted by each model and for each student. An average error, namely mean squared error (MSE), in prediction across all the individuals in the testing set was produced. This process was repeated 1000 times to generate a distribution of the MSEs for each model. We then chose one type of model, taking into consideration its variation in prediction, that showed the *best* performance on average in the testing set for the final prediction of ITEs. Note that the highlighted word “best” here and elsewhere in the paper does not suggest the best of all possible models, it only means the best amongst the models deployed, i.e., GLM, MLM, and RF. The GLM for prediction here differs from the earlier OLS model for interaction test, as the former has more variables than the latter in order to predict well and is less concerned about collinearity and the coefficient of a particular variable. GLM and MLM are the primary evaluation models in almost all EEF trials, it is natural to compare them with RF, which have excellent performances in prediction^40,43,51,56,57^.

Based on the Chess in Schools data, MLM has an average MSE of 0.42 and a standard deviation (*sd*) of 0.02, and those of GLM and RF are 0.49 $(sd=0.02)$ and 0.5 $(sd=0.02)$, respectively. The distributions of the prediction errors for the three types of models employed are illustrated in Fig. S14. We should choose MLM when the target of prediction is post-test score at individual level. Amongst the 1000 sets of parameters for MLM, the 854^th^ has the lowest out-of-sample prediction error, as reported in Table S2. That set of parameters should be used to make factual and counterfactual predictions for each individual. The factual and counterfactual datasets differ only in treatment status, where the factual values represent the real random assignment in the RCT, and their counterfactual values are the opposite of the actual random assignment. In other words, if a student was randomly assigned to the intervention group, the value for treatment indicator is 1, and its counterfactual value is 0, as if the student were assigned to the business-as-usual group.

The distribution of the 1000 prediction errors of MLM, as shown in Fig. S14, has some outliers. Choosing the best set of parameters with the lowest prediction error may produce biased results in the prediction. To take into consideration the variation in prediction errors, we examined three sets of parameters in MLM, one with the lowest prediction error (854^th^), the other two closest to half (792^th^) and one (805^th^) *sd* of the 1,000 MSEs above the set with the lowest error. Nevertheless, as reported in Table S2, the choice about which set to use for the prediction of post-test outcomes does not make much difference in the results. But when error distributions have extreme outliers in other studies, as shown in Figs. S15 and S16, the choice will make a substantial difference.

Given the data, we have one observed outcome, which we call Factual Outcome (FO), for each student. We can also use the best model to make two predictions for each individual, we call those predicted outcomes factual prediction (FP) and counterfactual prediction (CFP), with the former being the outcome predicted under the factual treatment condition a student actually allocated to, and the latter being the outcome predicted under the alternative treatment condition the student could have been allocated to. This means there are two ways to calculate ITEs, one being the difference between FO and CFP, and the other being FP and CFP. We assessed the performances of both approaches. Since we converted the outcome variable into *z*-scores, in either way, the ITEs computed are comparable to the reported effect sizes.

When ITEs were calculated as the differences between factual and counterfactual predictions (FP - CFP), they were constant or pre-determined by the chosen linear models. When we used the differences between the factual outcomes (FO) and counterfactual predictions (CFP) to calculate ITEs, the predictions reflected real-world uncertainties (the *sds* are much larger in the FO - CFP rows of Table S2).

For the above reasons, we employed RF to predict ITEs, as they were less deterministic and more responsive to individual differences than the other two types of linear models. However, when the target of prediction was a potential outcome, rather than the difference of two potential outcomes, the ranges of ITEs were too wide, as shown in Fig. 2A. This means the chosen RF have not learned enough about the data, particularly, the relationships between the differences we are interested in and other covariates. This empirically explains the need to focus on the effects rather than the prediction of potential outcomes alone for causal inference^40,43,58^.

In order to reduce the variation in the prediction of ITEs, we re-trained the RF by fine-tuning the hyperparameters of the algorithm using its out-of-bag (OOB) error estimates. The speed of the R package, ranger^59^, allowed us to conduct a random grid search on the three main hyperparameters: *mtry*—the number of variables sampled for splitting at each node; sample fraction – the proportion of the full dataset provided to each tree for training (through bootstrapping); and minimum node size – the minimum number of data points in each terminal node acting as a regulariser on each tree. To increase our confidence in selecting an optimal set of hyper-parameters in what is an inherently noisy system (due to the random sampling of the RF algorithm), we used heterogeneous Gaussian process regression^60^ to smooth out the noise, and selected the hyperparameters that minimised the upper confidence bound on OOB error. As Fig. 2A shows, the ITEs from the re-trained RF have a narrower spread than those from the RF mentioned earlier.

## Supplementary Information

Supplementary Information 1.

## Data Availability

As part of their funding scheme, the EEF requires all evaluation teams to submit their data to a central archive, which is managed by FFT Education and held by the ONS within their Secure Research Service. FFT provided us with 48 unique data extracts from large-scale RCTs of varied designs. The data were linked with the National Pupil Database in England, but de-identified at pupil and school levels. The 48 datasets that support the findings of this study are available from the EEF, but restrictions apply to the availability of these data. Please refer to this link about access to the data: https://www.ons.gov.uk/aboutus/whatwedo/statistics/requestingstatistics/approvedresearcherscheme or contact FFT Education (via this link https://fft.org.uk/about-fft/) to request access to the data. Because the data ownership lies with the EEF/FFT/ONS, they make ultimate decisions on who to grant access.
