# Supplementary material for: Uncovering individualised treatment effects for educational trials
Source: Sci Rep. 2024 Sep 30;14:22606. doi: 10.1038/s41598-024-73714-z (PMC11442981; doi:10.1038/s41598-024-73714-z)
Supplement: Supplementary file 1 — Supplementary Information 1. [file 41598_2024_73714_MOESM1_ESM.pdf]

Supplementary Information for

Uncovering Individualised Treatment Effects for Educational Trials

ZhiMin Xiao, Oliver Hauser, Charlie Kirkwood, Daniel Z. Li, Tamsin Ford, and Steve Higgins

To whom correspondence may be addressed. Email: zhimin.xiao@essex.ac.uk

This PDF file includes:

Supplementary text
Figs. S1 to S16
Tables S1 to S2
SI References

Supporting Information Text

1. The Data Archive and the Pre-Specified Subgroups

To enable further exploration and development, the Education Endowment Foundation (EEF) in England has created a data archive of all the trials they fund. As the charity intends to award as much as £200 million by 2026, the archive will keep growing and gradually be linked with the National Pupil Database (NPD) in England and housed in the Office for National Statistics Secure Research Service (SRS). For the time being, the FFT Education Ltd is responsible for the curation of the archive. Since individual evaluation teams of EEF projects submit their data to the FFT, which then process the data one more time and link them with the NPD, the archive dataset is relatively well-structured. However, multiple pre-processing of data does pose some challenges to the reproducibility of research findings (1, 2). For instance, project specific data the FFT receive may not be raw data, as evaluation teams sometimes need to make decisions on which observations to exclude or include in their final analysis, which may be complete case analysis or involve imputation of missing data in multiple ways. The reconciliation of sample sizes through several stages of data pre-processing and analysis is challenging enough and sometimes leads to inconsistent sample sizes in the archive data and those in evaluation reports.

When it comes to subgroup analysis of FSM pupils, the procedure becomes even more challenging, partly because not all evaluation teams reported subgroup sample sizes, even though they were required to estimate an intervention effect for the subgroup. But the problem also stems from a conceptual difficulty, as it is not always easy to identify FSM students at the beginning of an intervention. As a result, the archive dataset has four binary variables related to FSM status. In all cases, one means "eligible" for FSM, and zero suggests "Not eligible". The first one is `Pupil_FSM`, which indicates FSM eligibility at the beginning of an intervention. The second is `Pupil_FSM6`, which indicates FSM eligibility in the previous six years from the beginning of an intervention. These two variables are project specific because they are not extracted from the NPD. The FFT links pupils in the interventions with records in the NPD, so the data archive has two extra FSM variables called `NPD_EVERFSM_6` and `NPD_EVERFSM_ALL`. The former means "Recorded as eligible for free school meals in the last 6 years (based on Spring Census only up to 2011/12)", and the latter is described as "Recorded as eligible for free school meals in any Spring Census up to the current year". As noted above, even when a subgroup is pre-specified at the conceptual level, the actual definition can vary at the operational level, not to mention the possibility to differentiate pupils who are eligible for FSM from those who are eligible *but* have not claimed FSM. Eventually, we choose `NPD_EVERFSM_6` to define FSM and Non-FSM subgroups in the 48 projects considered and listed in Table S1 for two reasons. First, it has the least amount of missing data. Second, the FFT recommended this variable after we consulted them.

2. Conventional Approaches to Subgroup Analysis and Effect Estimation

Following some established approaches to subgroup analysis in the evaluation of EEF trials, we conduct treatment-FSM interaction tests and estimate separate effect sizes for both FSM and Non-FSM subgroups. The interaction test in our analysis is an Ordinary Least Squares (OLS) model with a treatment-FSM interaction term plus pre-test, a baseline measure available in almost all EEF trials. For each outcome, we obtain the sample size used for the test and the p -value associated with the interaction term. The functional form of the test is defined below (3):

$$Y_i = \beta_0 + \beta_1 t_i + \beta_2 pret_i + \beta_3 fsm_i + \beta_4 t_i \times fsm_i + \epsilon_i,$$

where Y_i is post-test results of individual students. t_i is treatment indicator with a value of 0 for control or 1 for intervention in a two-arm trial, and $pret_i$ is pre-test. $\epsilon_i \sim N(0, \sigma^2)$ is the residual component of the model (2).

To derive effect size estimates within the subgroups of FSM and Non-FSM students, we employ a multilevel model (MLM) and implement it using an `R` package called `eefAnalytics` (2) specifically developed to estimate effect sizes for RCTs of varied designs. To facilitate comparability of effect sizes from across the studies, the model controls for a project specific baseline measure of pre-test in the fixed component and allows the random part of the hierarchical linear model to vary across schools. The two subgroup effect estimates are then presented together with the overall effect sizes independent evaluation teams reported.

When average pupil attainment varies considerably from school to school and/or there are unequal samples per school in an RCT, MLM is recommended to estimate the weighted average across schools as well as the variances that occur across pupils (level 1) and schools (level 2) (4, 5).

To build the first level of the MLM, we use the equation below:

$$Y_{ij} = \beta_{0j} + \beta_{1j} t_{ij} + \beta_{2j} pret_{ij} + r_{ij},$$

the continuous outcome variable Y_{ij} represents post-test result of student i in school j, where $j = 1, 2, \cdots, M$ and $i = 1, 2, \cdots, n_j$. M is the number of schools in a trial, n_j is the number of pupils per school, and $r_{ij} \sim N(0, \sigma^2_{within})$

Table S1. EEF project information: 84 outcomes from 48 projects, with links to full official reports.

Project No.	Archive outcome label	Full EEF project title	Link
1	abral, abraNI	ABRA: Online Reading Support	bit.ly/2P4p2if
2	alph	Tutoring with Alphie	bit.ly/2PmlOW8
3	AOMT	Affordable Online Maths Tuition	bit.ly/2sa7w34
4	ar	Accelerated Reader	bit.ly/2Yvkzbu
5	aspl, aspm	Act, Sing, Play	bit.ly/2PwyhHb
6	cbks, cbks+	Chatterbooks	bit.ly/2P38reO
7	chess	Chess in Schools	bit.ly/38nltdq
8	cl	Catch Up Literacy	bit.ly/2LDwA9E
9	cmpe, cmpm, cmte, cmtm	Changing Mindsets	bit.ly/2YvlkkQ
10	dsm3, dsm5	Durham Shared Maths	bit.ly/36mUHRX
11	efm, efr, efw	Effective Feedback	bit.ly/36mLKI3
12	ffe, ffm	Future Foundations	bit.ly/36lUrCO
13	fs	Fresh Start	bit.ly/2LzT2Af
14	gfw	Grammar for Writing	bit.ly/2rxnRyK
15	hh	Hampshire Hundreds	bit.ly/2scmaqs
16	impl, impn	Improving Numeracy and Literacy	bit.ly/2RABJ5W
17	ipmee, ipmem, ipmfe, ipmfm	Increasing Pupil Motivation	bit.ly/2LDBiEc
18	iwq	Improving Writing Quality	bit.ly/2RzUTZW
19	lit	LIT Programme	bit.ly/355nquh
20	ltss	Let's Think Secondary Science	bit.ly/2YxS0u2
21	mbk1m, mbk1r, mbk1w, mbk2r	Magic Breakfast	bit.ly/2PwwRML
22	mms	Mathematics Mastery Secondary	bit.ly/38lpr7E
23	p4cm, p4cr, p4cw	Philosophy for Children	bit.ly/2YxSksK
24	paicM, paicR, pauicM, PauicR	Parent Academy	bit.ly/38iIgs2
25	pale, palm	Physically Active Lessons	bit.ly/2Ywwx4u
26	pbcp	Perry Beeches Coaching Programme	bit.ly/2scoiys
27	teepe, teepm	Teacher Effectiveness Enhancement	bit.ly/2rsBQWA
28	plce, plcm	Powerful Learning Conversations	bit.ly/38vO7KO
29	pr7, pr9	Paired Reading	bit.ly/2sSXROH
30	quest	Quest	bit.ly/2P2pGge
31	rflEdM, rflEdR	ReflectEd	bit.ly/2RGwy4q
32	rfr	Rhythm for Reading	bit.ly/36t4w0P
33	rp	Rapid Phonics	bit.ly/2LAUgeA
34	rti	Response to Intervention	bit.ly/344VJ3x
35	sar	Summer Active Reading	bit.ly/2YuA8QC
36	shineE, shineM	SHINE in Secondaries	bit.ly/2P5DR46
37	sor	Switch-on Reading	bit.ly/340oRsF
38	spksA, spksL, spksW	SPOKES	bit.ly/2P5Eivg
39	tdts	Thinking, Doing, Talking Science	bit.ly/345ewLK
40	ttpe, ttpm, ttse, ttsm	Tutor Trust Primary and Secondary	bit.ly/354Y6Vj
41	text	TextNow	bit.ly/356VqX0
42	tfl	Talk for Literacy	bit.ly/36jHppk
43	TotT	Talk of the Town	bit.ly/357kUnd
44	txtpE, txtpM, txtpS	Texting Parents	bit.ly/36jjmqk
45	uos	Units of Sound	bit.ly/2t2DEWX
46	ve	Vocabulary Enrichment	bit.ly/2P2sgTs
47	wwr	Word and World Reading	bit.ly/3578ewu
48	YSAE, YSAM	Youth Social Action	bit.ly/2sW9ZhT

captures student level differences, or within-school variance, in post-test results around school means. t_{ij} and $pret_{ij}$ are the observed treatment status and pre-test score for student i in school j.

At the second level, the model is specified as:

$$\beta_{0j} = \gamma_{00} + u_{0j}, \;\; \beta_{1j} = \gamma_{10}, \;\text{and}\; \beta_{2j} = \gamma_{20},$$

where school average, β_{0j}, is a function of the grand-mean in post-test, γ_{00}, plus school-level residuals around that grand-mean, which are modelled as $u_{0j} \sim N(0, \sigma^2_{between})$, where $\sigma^2_{between}$ represents between-school variance. The equations at level two assume average attainment varies across schools, but consider average treatment and pre-test

effects constant or fixed at γ_{10} and γ_{20} respectively, hence the absence of u_{1j} and u_{2j}.

Substituting level two equations into level one results in a MLM that simultaneously estimates the weighted effect of intervention across schools as well as the different sources of variation. The combined model is therefore constructed as:

$$Y_{ij} = \gamma_{00} + \gamma_{10} t_{ij} + \gamma_{20} pret_{ij} + r_{ij} + u_{0j},$$

where γ_{10} is the estimated mean difference in intervention effect between the intervention and control schools, t_{ij} is a dummy variable coded as 1 for pupils in intervention schools and 0 for pupils in control schools, and $u_{0j} \sim N(0, \sigma^2_{between})$ captures the variation between schools. r_{ij} and u_{0j} are assumed to be independent and respectively capture within- and between-school variances.

Effect size estimation under the MLM depends on which source of variation is used. Using within-school variance, it is calculated as:

$$\frac{\gamma_{10}}{\sqrt{\sigma^2_{within}}}.$$

However, effect size based on within- or between-school variance may be inflated (2). We therefore choose total variance, and the effect size is calculated as:

$$\frac{\gamma_{10}}{\sqrt{\sigma^2_{within} + \sigma^2_{between}}}.$$

The linear models employed to detect and estimate separate effect sizes are common approaches in the evaluation of EEF trials. We adopt and apply the same models consistently across all the studies investigated. We use an OLS rather than a MLM to compute p-values because the former is less conservative than the latter and the comparison aims to see what the results will be like when the interaction test is most likely to detect effect heterogeneity. We are acutely aware that people from different disciplines often adopt different ways to compute interaction test p-values, which only magnifies the point we want to highlight – current approaches to subgroup analyses are inconsistent, potentially leading to mixed results.

A. Results of the interaction tests. In total, we examine 84 outcomes from 48 projects, which are largely independent projects designed and evaluated by independent teams in different years. To facilitate comprehension of the results, we highlight a few studies in Fig. S1, which, as in Figs. S2 to S13, reports three effect size estimates, their associated sample sizes and 95% confidence intervals. The first, for reference only, is the overall effect size (`Overall`) every EEF project reports for all the students involved. The other two are separate estimates produced by us for Non-FSM (`nFSM`) and FSM (`FSM`) students. We also report the p-values from the treatment-FSM interaction tests and their sample sizes in column `p`. The outcomes are then sorted in ascending order of those p-values.

Note that the sample sizes evaluators reported for their overall effect sizes may not always be equal or even close to the sums of FSM and Non-FSM pupils in our analyses. While this discrepancy arises partly because the FSM variables evaluators used are sometimes different from the one we use, it also stems from the differences in model specification and the ways in which missing data are dealt with. As a result, the overall effect sizes independent evaluation teams reported do not have to be in the middle of the two subgroup estimates, as their models and sample sizes are sometimes different from ours.

Now let's see what the interaction tests have to reveal about the estimates for the two subgroups. `txtpS` in Fig. S1 is an outcome for science from an intervention called Texting Parents. The p-value from the interaction test is almost zero, but we do not see a significantly big difference in point estimates for Non-FSM $-0.06(-0.30, 0.17)$ and FSM $-0.09(-0.37, 0.19)$ students. The overall effect size the evaluation team reported is $-0.01(-0.05, 0.02)$, and neither of the subgroup estimates is statistically significant at the standard significance level of $\alpha = 0.05$. We can find the same pattern in other outcomes, such as `teepe` of the same figure.

When a p-value is relatively large, such as `cbks` where $p = 0.127$, the two point estimates for Non-FSM and FSM pupils are 0.09 and -0.23 respectively, a typical case of "qualitative interaction" (6), meaning the point estimates not only change in magnitude, but also in direction. The p-value from the interaction test for `mbk2r` is 0.184, and the project has a non-significant overall effect size of $0.10(-0.06, 0.26)$, but a statistically significant one for Non-FSM students at $0.22(0.02, 0.42)$ and a much smaller and non-significant effect for FSM students at $0.07(-0.10, 0.23)$. This is an example of "quantitative interaction" (6), where the effect sizes for the subgroups do not differ in direction, but a lot in magnitude.

In earlier cases of Figs. S2 to S13, the p-values are small, we can find differential effects for the two subgroups. As we move to later figures, the differences between the two subgroup estimates become smaller and smaller. But this is not always the case, for instance, `AOMT` and `spksW`, also in Fig. S1, still exhibit substantial differences in point

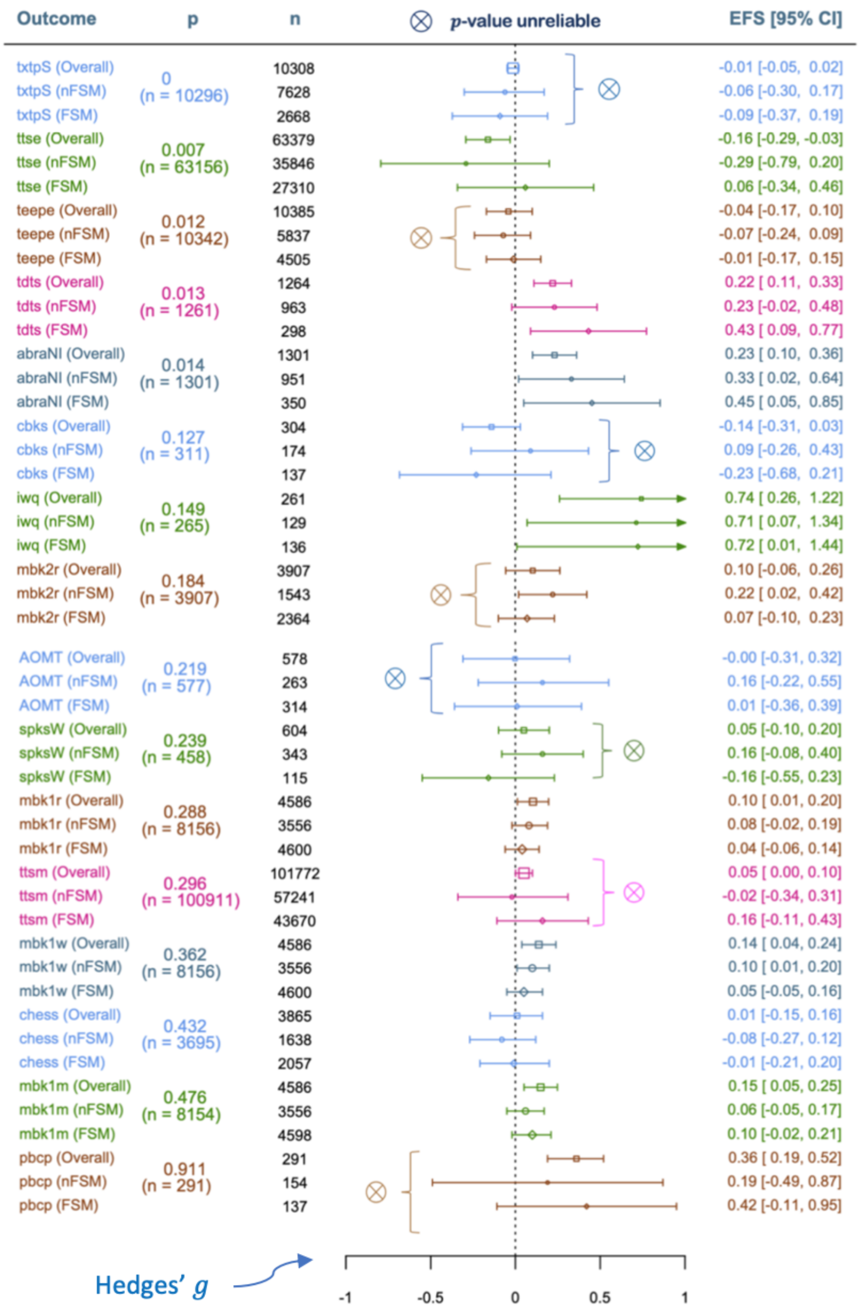

Fig. S1. ***p*-values from treatment-FSM interaction tests can be *unhelpful* to detect effect heterogeneity: it fails to show a signal when there is one, and indicates one when there is not.** `txtpS` – science in Texting Parents. `ttse` – English in Tutor Trust Secondary; `lit` – LIT; `teepe` – English in Teacher Effectiveness Enhancement Programme; `tdts` – Thinking, Doing, Talking Science; `abraNI` – Non-ICT in ABRA; `cbks` – Chatterbooks; `iwq` – Improving Writing Quality; `mbk2r` – KS2 reading in Magic Breakfast; `AOMT` – Affordable Online Maths Tuition; `spksW` – word ID in SPOKES; `mbk1r` – KS1 reading in Magic Breakfast; `ttsm` – maths in Tutor Trust Secondary; `mbk1w` – KS1 writing in Magic Breakfast; `chess` – Chess in Schools; `mbk1m` – KS1 maths in Magic Breakfast; `pbcp` – Perry Beeches Coaching Programme.

estimates, a quantitative interaction in the former and a qualitative one in the latter, where the p-value is by no means small at 0.239. As we move to cases that have larger and larger p-values, we can still find, for instance in `ttsm` where the p-value is as large as 0.296, a qualitative interaction, which is considered rare in the literature (6). And in

`pbcp`, the p-value is 0.911, but the quantitative interaction has a difference in point estimates of 0.23, which is larger than most overall effect sizes in all of the trials the EEF has funded to date (7).

There is no need to list all the outcomes that show how *unhelpful* an interaction test can be. We emphasise the word "unhelpful" because there are also many cases where a p-value is helpful. In the cases that have larger p-values, the differences between the two subgroup estimates are narrower in general.

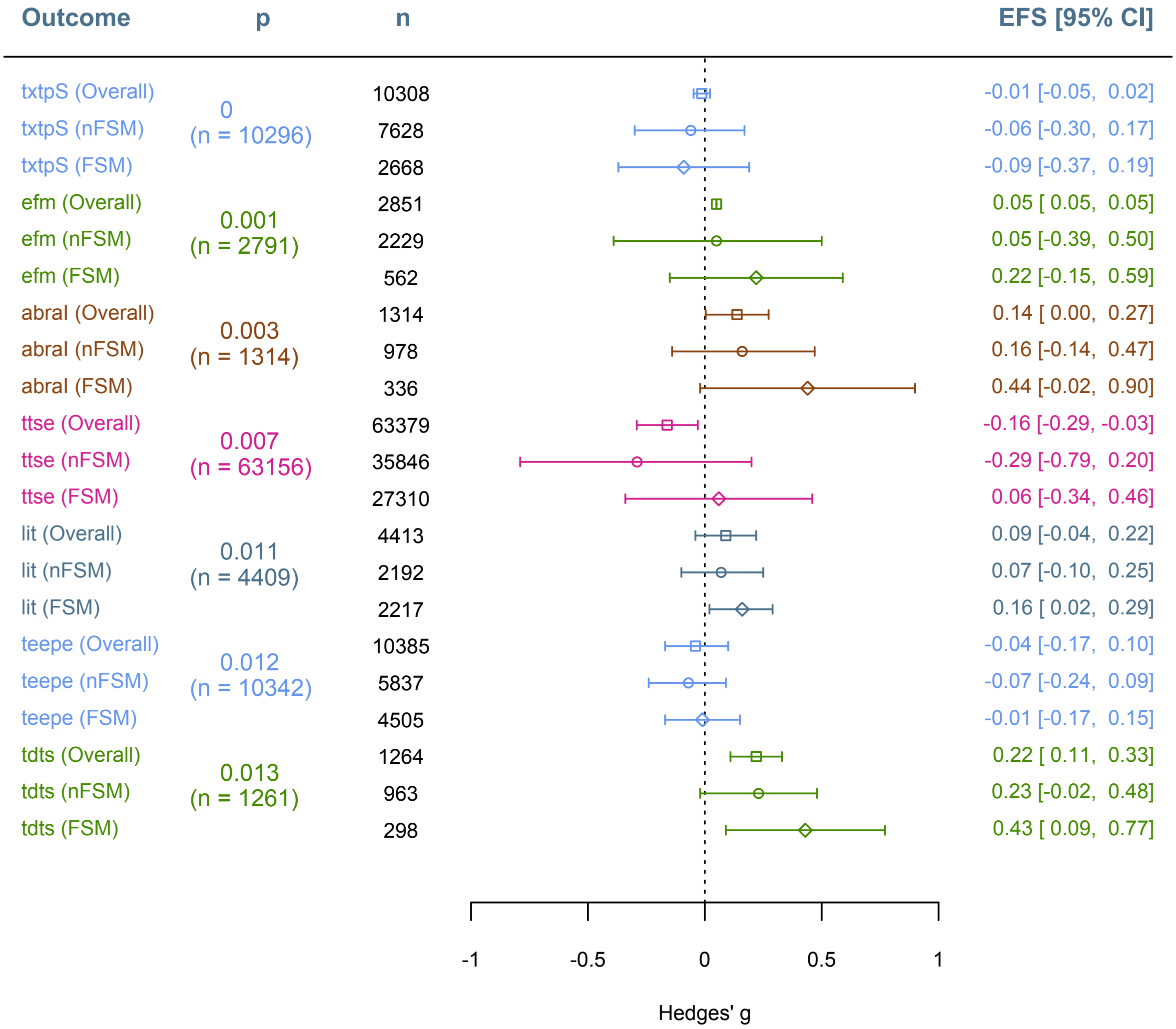

Fig. S2. Outcomes ordered by ascending p-values from FSM-Treatment interaction tests. Each outcome has three effect estimates, the first is a study's overall effect size (`Overall`), the second and third are our estimates for Non-FSM (`nFSM`) and FSM (`FSM`) pupils, respectively. `txtpS` – science in Texting Parents; `efm` – maths in Effective Feedback; `abraI` – ICT in ABRA; `ttse` – English in Tutor Trust Secondary; `lit` – LIT; `teepe` – English in Teacher Effectiveness Enhancement Programme; `tdts` – Thinking, Doing, Talking Science.

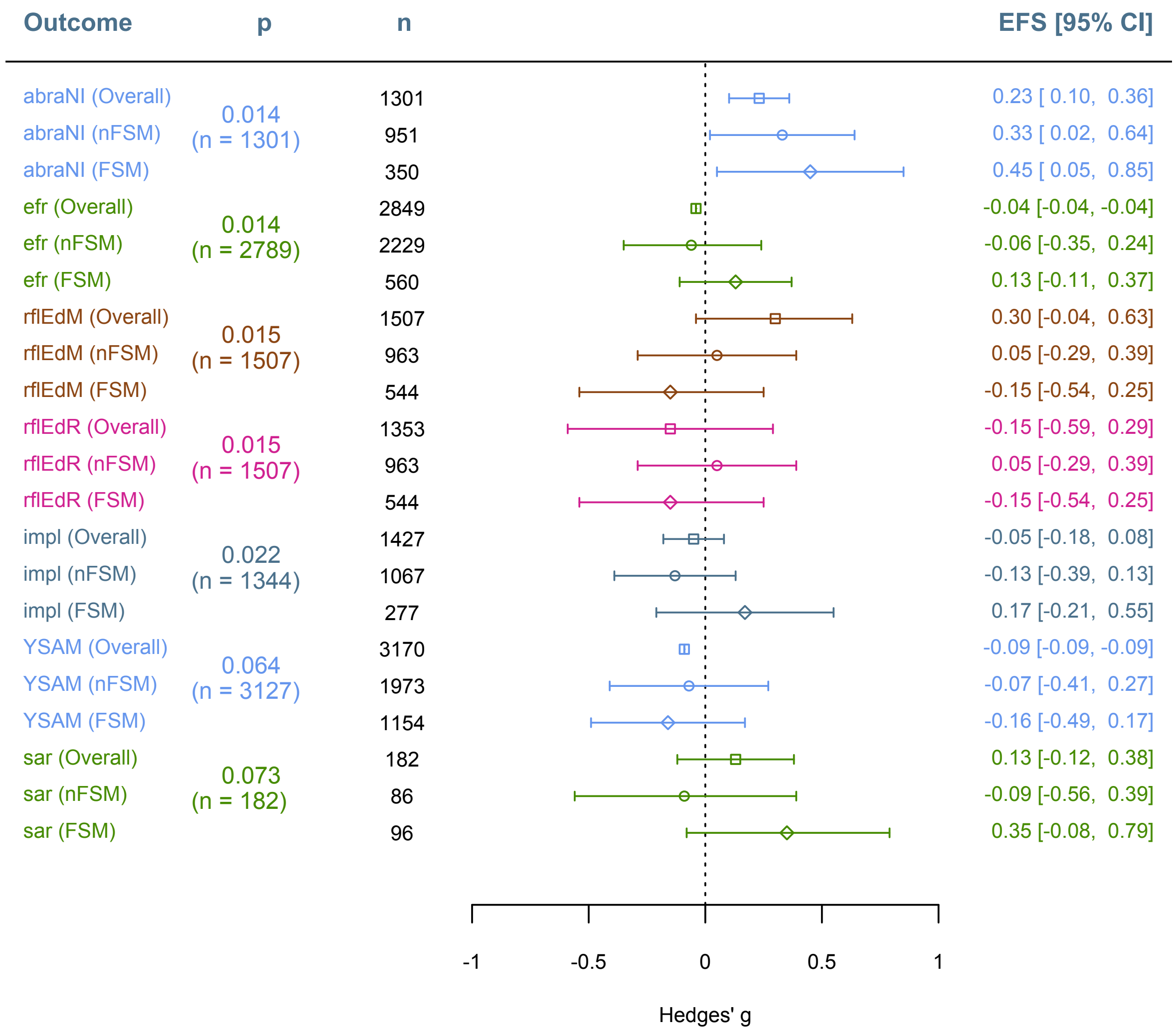

Fig. S3. Outcomes ordered by ascending p-values from FSM-Treatment interaction tests. `abraNI` – Non-ICT in ABRA; `efr` – reading in Effective Feedback; `rflEdM` – maths in ReflectEd; `rflEdR` – reading in ReflectEd; `impl` – literacy in Improving Numeracy and Literacy; `YSAM` – maths in Youth Social Action; `sar` – Summer Active Reading.

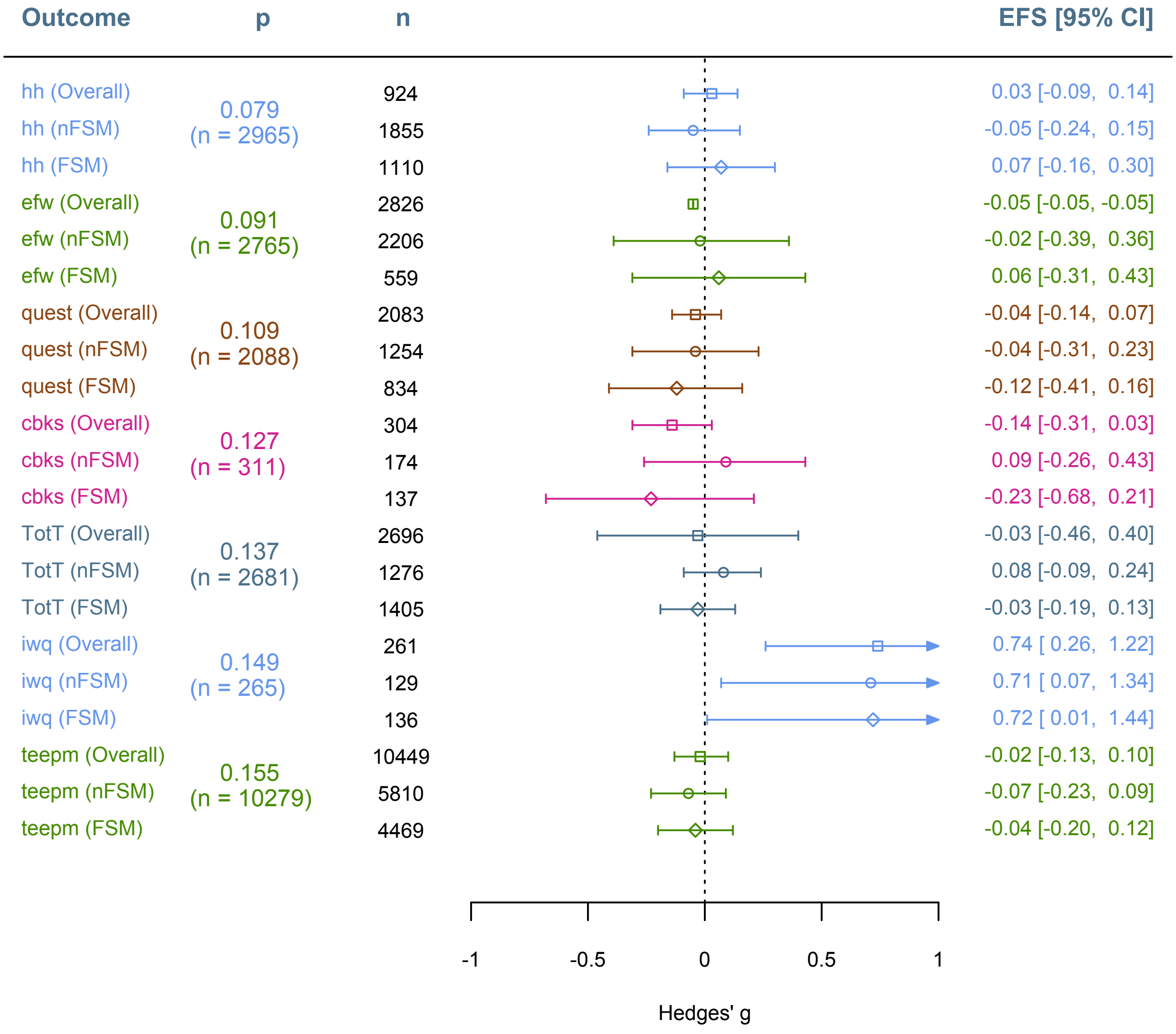

Fig. S4. Outcomes ordered by ascending p-values from FSM-Treatment interaction tests. `hh` – Hampshire Hundreds; `efw` – writing in Effective Feedback; `quest` – Quest; `cbks` – Chatterbooks; `TotT` – Talk of the Town; `iwq` – Improving Writing Quality; `teepm` – maths in Teacher Effectiveness Enhancement Programme.

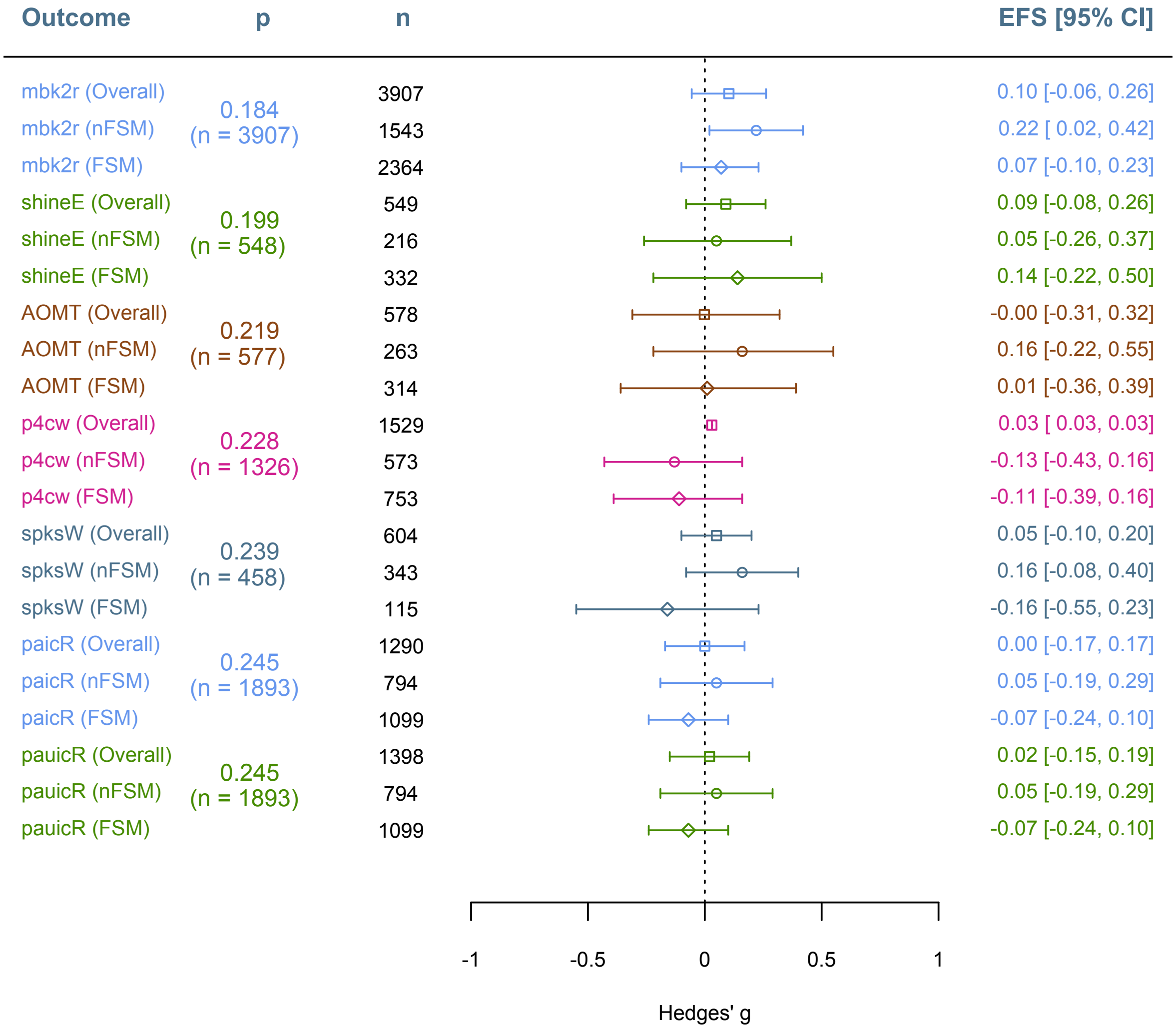

Fig. S5. Outcomes ordered by ascending p-values from FSM-Treatment interaction tests. `mbk2r` – KS2 reading in Magic Breakfast; `shineE` – English in SHINE in Secondaries; `AOMT` – Affordable Online Maths Tuition; `p4cw` – writing in Philosophy for Children; `spksW` – word ID in SPOKES; `paicR` – reading in Parenting Academy (incentivised); `pauicR` – reading in Parenting Academy (unincentivised).

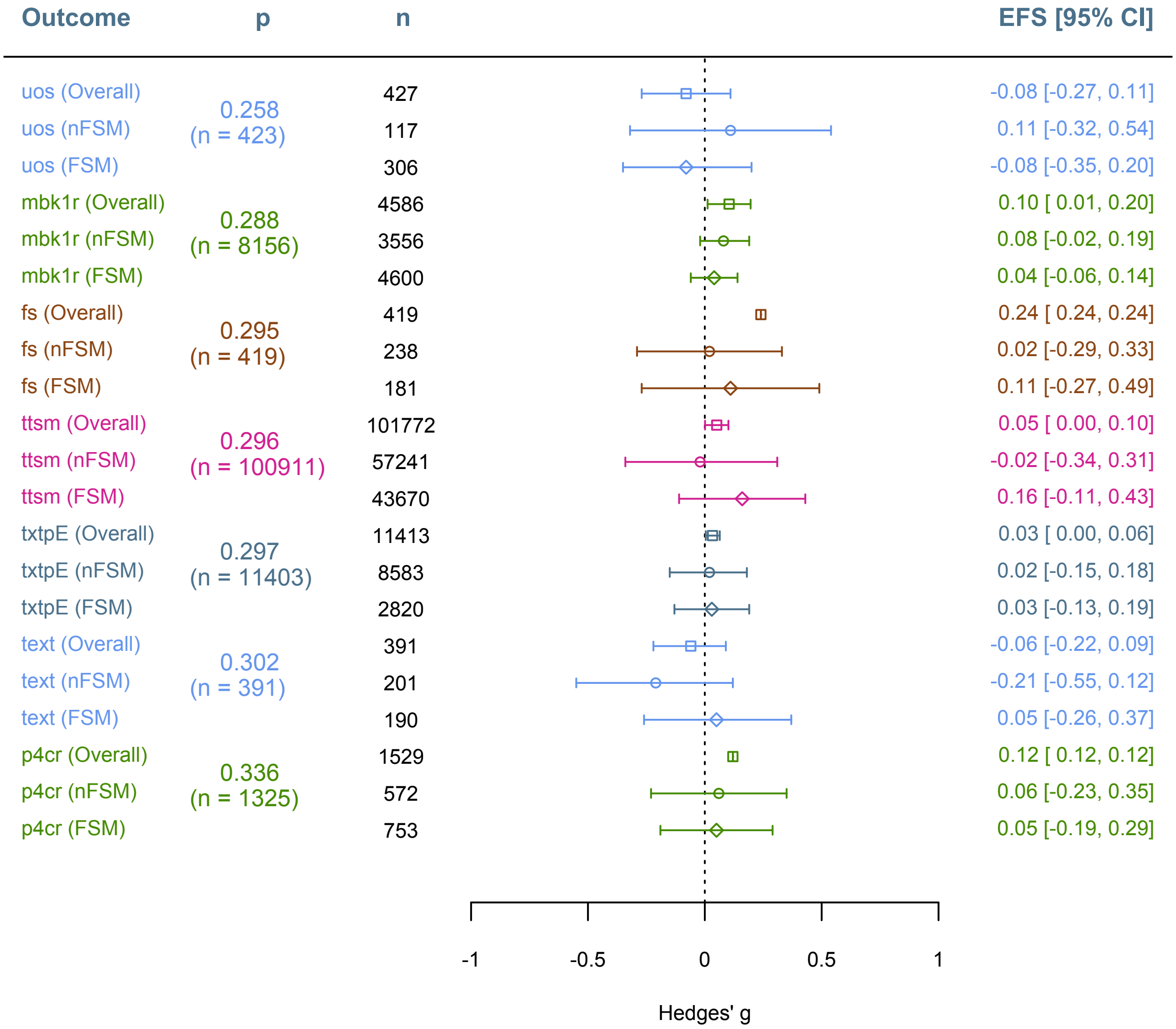

Fig. S6. Outcomes ordered by ascending p-values from FSM-Treatment interaction tests. `uos` – Units of Sound; `mbk1r` – KS1 reading in Magic Breakfast; `fs` – Fresh Start; `ttsm` – maths in Tutor Trust Secondary; `txtpE` – English in Texting Parents; `text` – TextNow; `p4cr` – reading in Philosophy for Children.

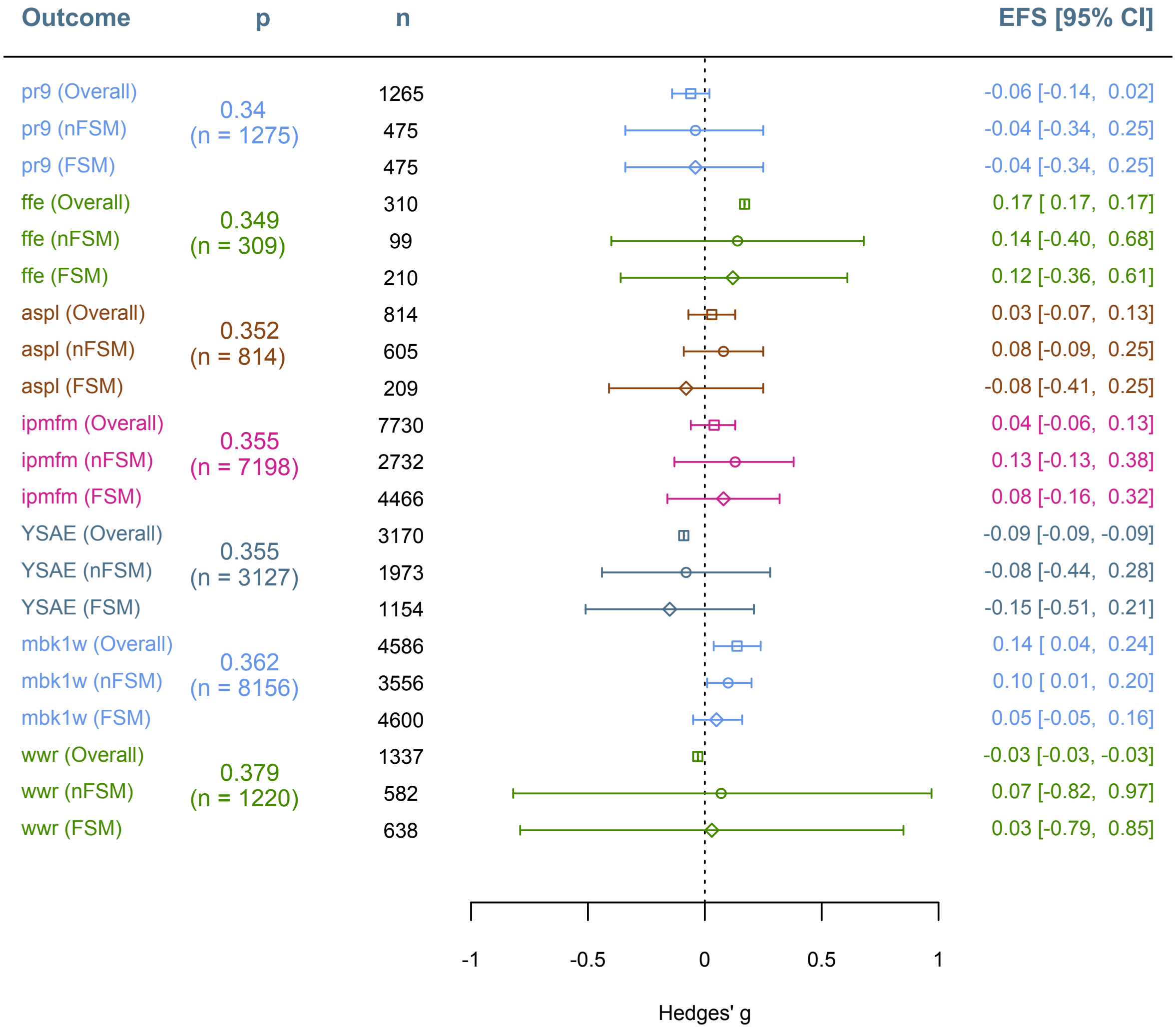

Fig. S7. Outcomes ordered by ascending p-values from FSM-Treatment interaction tests. `pr9` – Paired Reading for Year 9; `ffe` – English in Future Foundations; `aspl` – literacy in Act, Sing, Play; `ipmfm` – maths in Increasing Pupil Motivation (finance); `YSAE` – English in Youth Social Action; `mbk1w` – KS1 writing in Magic Breakfast; `wwr` – Word and World Reading.

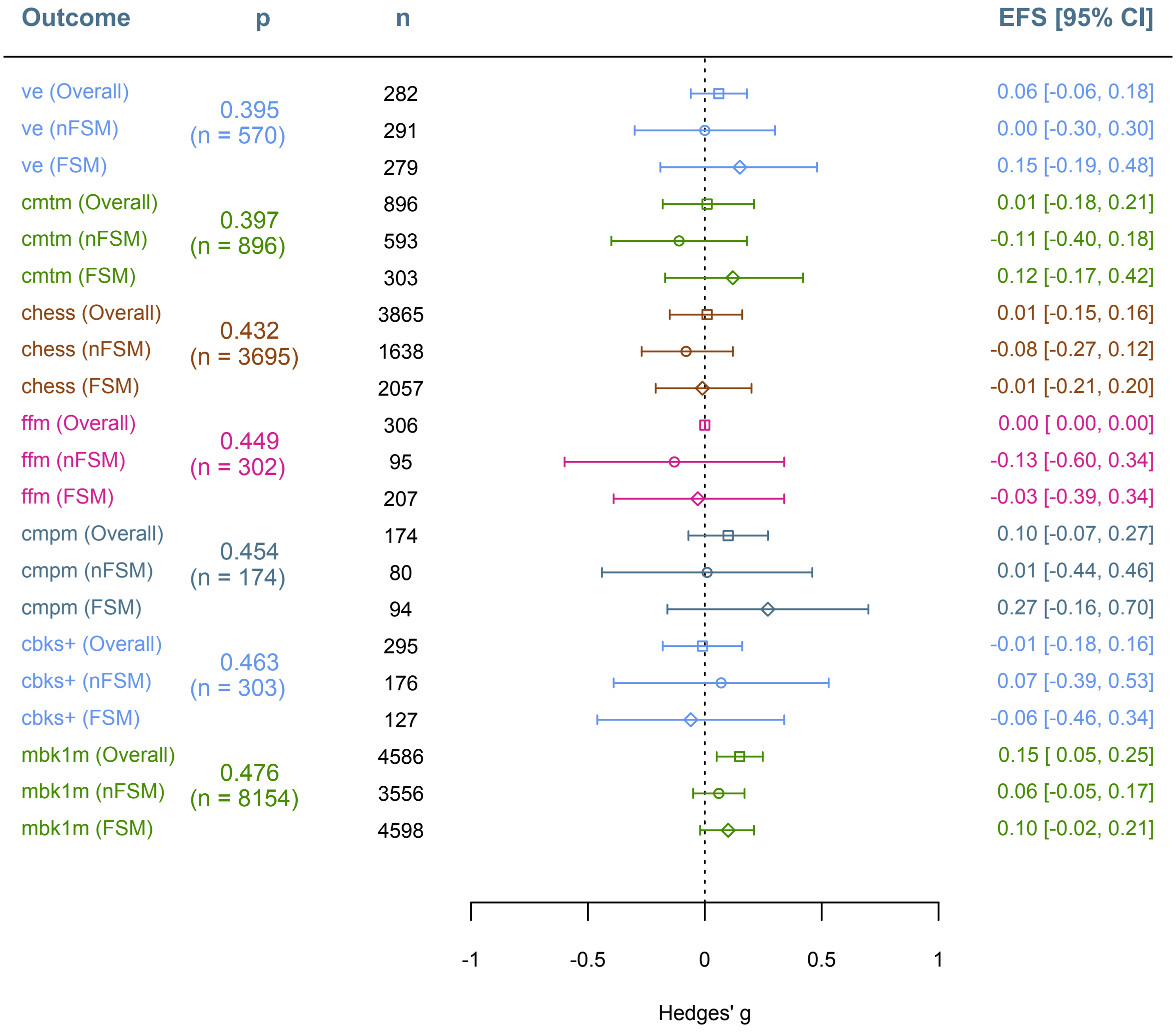

Fig. S8. Outcomes ordered by ascending p-values from FSM-Treatment interaction tests. `ve` – Vocabulary Enrichment; `cmtm` – maths in Changing Mindsets (teacher training); `chess` – Chess in Schools; `ffm` – maths in Future Foundations; `cmpm` – maths in Changing Mindsets (pupil workshops); `cbks+` – Chatterbooks Plus; `mbk1m` – KS1 maths in Magic Breakfast.

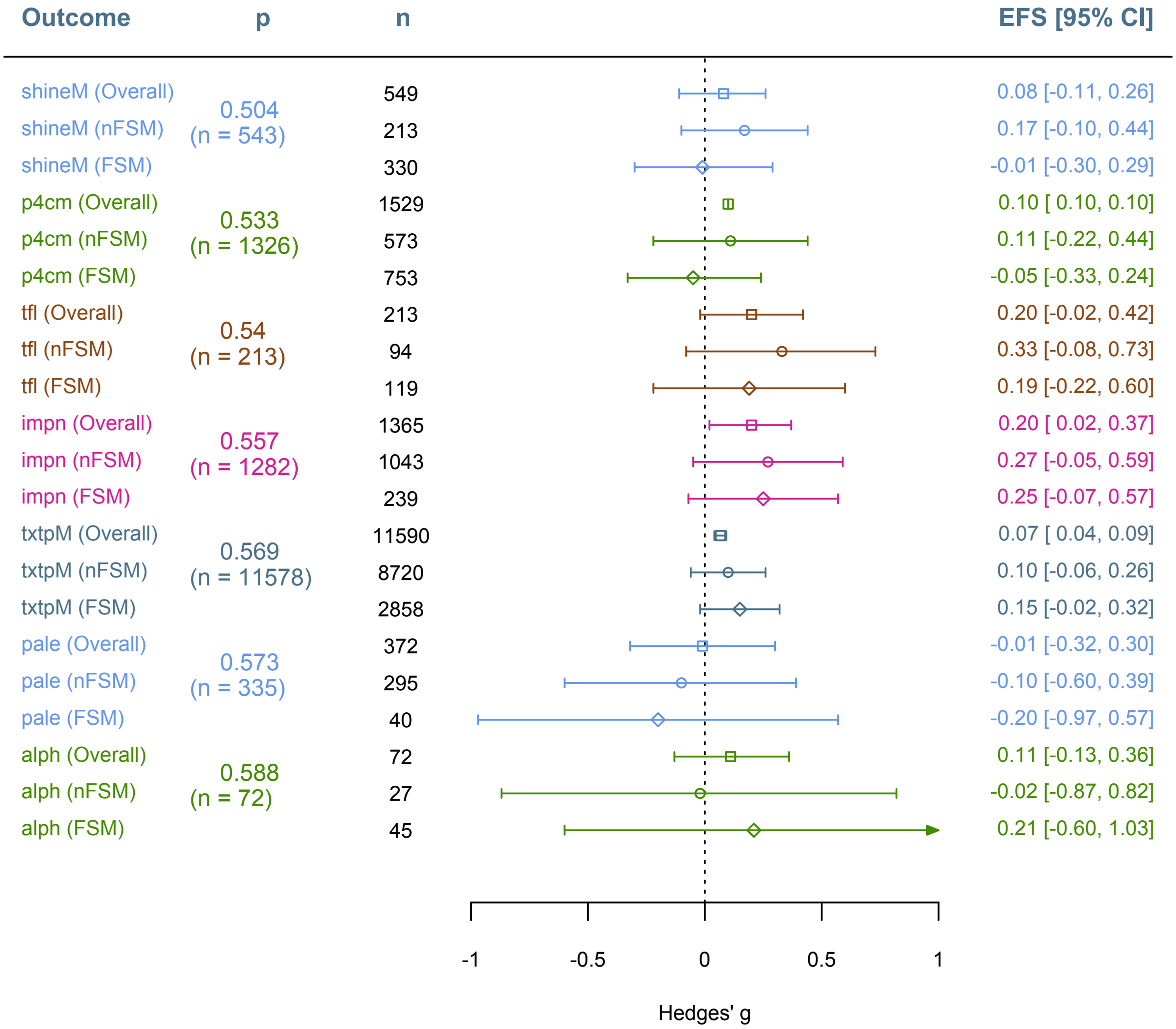

Fig. S9. Outcomes ordered by ascending p-values from FSM-Treatment interaction tests. `shineM` – maths in SHINE in Secondaries; `p4cm` – maths in Philosophy for Children; `tfl` – Talk for Literacy; `impn` – numeracy in Improving Numeracy and Literacy; `txtpM` – maths in Texting Parents; `pale` – English in Physically Active Lessons; `alph` – Tutoring with Alphie.

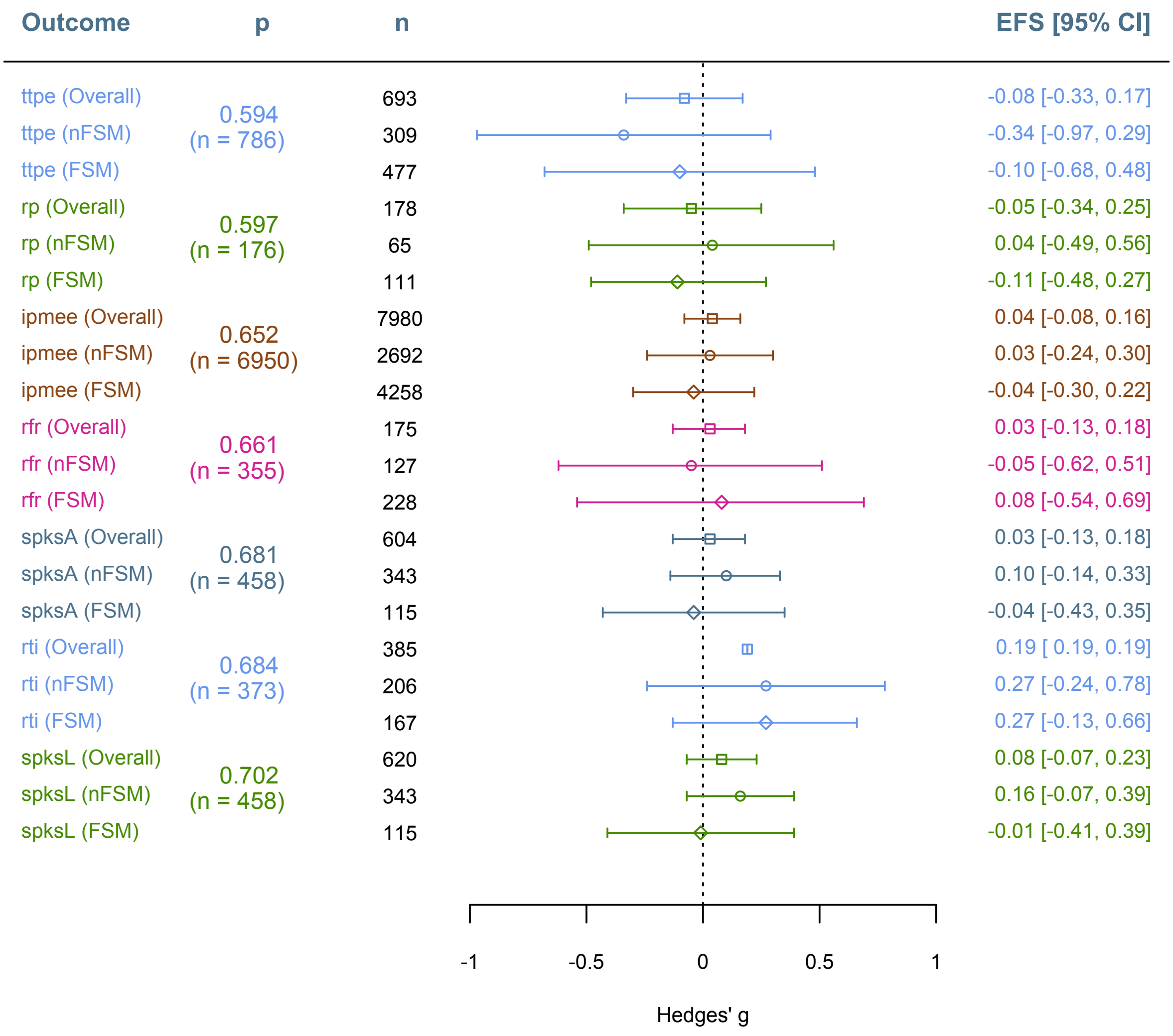

Fig. S10. Outcomes ordered by ascending p-values from FSM-Treatment interaction tests. `ttpe` – English in Tutor Trust Primary; `rp` – Rapid Phonics; `ipmee` – English in Increasing Pupil Motivation (event); `rfr` – Rhythm for Reading; `spksA` – Word Attack in SPOKES; `rti` – Response to Intervention; `spksL` – Letter ID in SPOKES.

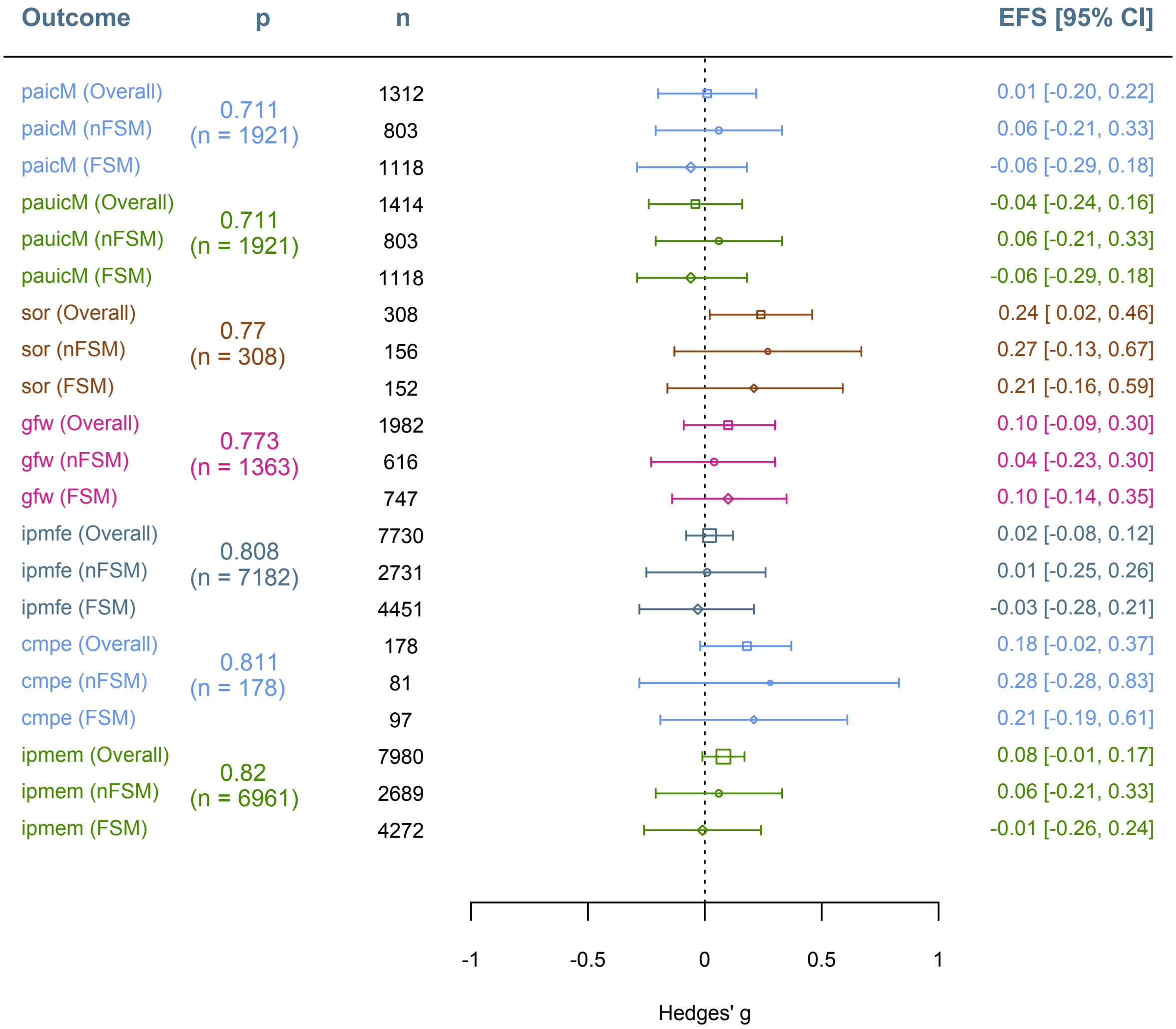

Fig. S11. Outcomes ordered by ascending p-values from FSM-Treatment interaction tests. `paicM` – maths in Parenting Academy (incentivised); `pauicM` – maths in Parenting Academy (unincentivised); `sor` – Switch-on Reading; `gfw` – Grammar for Writing; `ipmfe` – English in Increasing Pupil Motivation (finance); `cmpe` – English in Changing Mindsets (pupil workshops); `ipmem` – maths in Increasing Pupil Motivation (event).

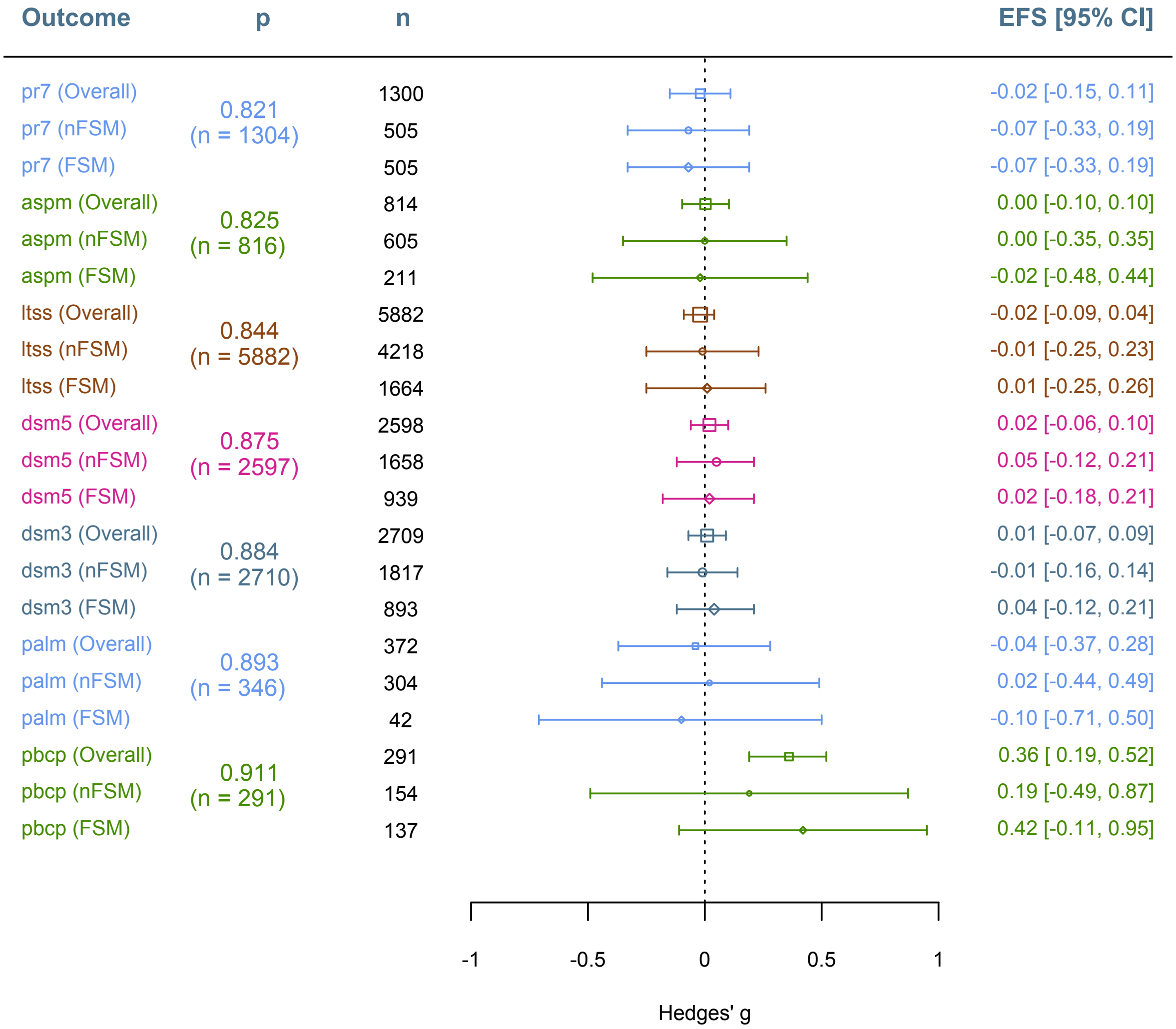

Fig. S12. Outcomes ordered by ascending p-values from FSM-Treatment interaction tests. `pr7` – Paired Reading (year 7); `aspm` – maths in Act, Sing, Play; `ltss` – Let's Think Secondary Science; `dsm5` – Durham Shared Maths (year 5); `dsm3` – Durham Shared Maths (year 3); `palm` – maths in Physically Active Lessons; `pbcp` – Perry Beeches Coaching Programme.

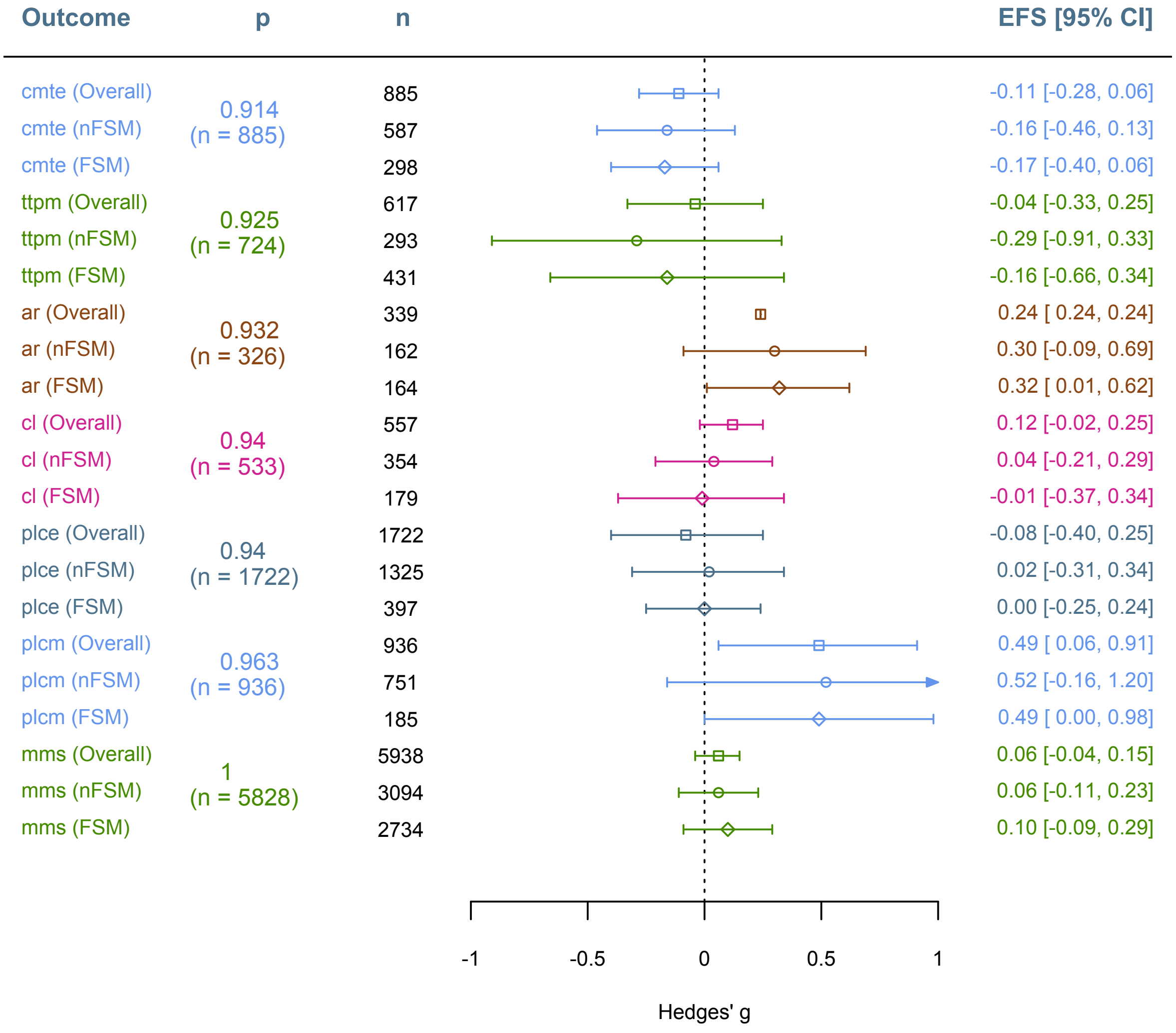

Fig. S13. Outcomes ordered by ascending p-values from FSM-Treatment interaction tests. `cmte` – English in Changing Mindsets (teacher training); `ttpm` – maths in Tutor Trust Primary; `ar` – Accelerated Reader; `cl` – Catch up Literacy; `plce` – English in Powerful Learning Conversations; `plcm` – maths in Powerful Learning Conversations; `mms` – Mathematics Mastery Secondary.

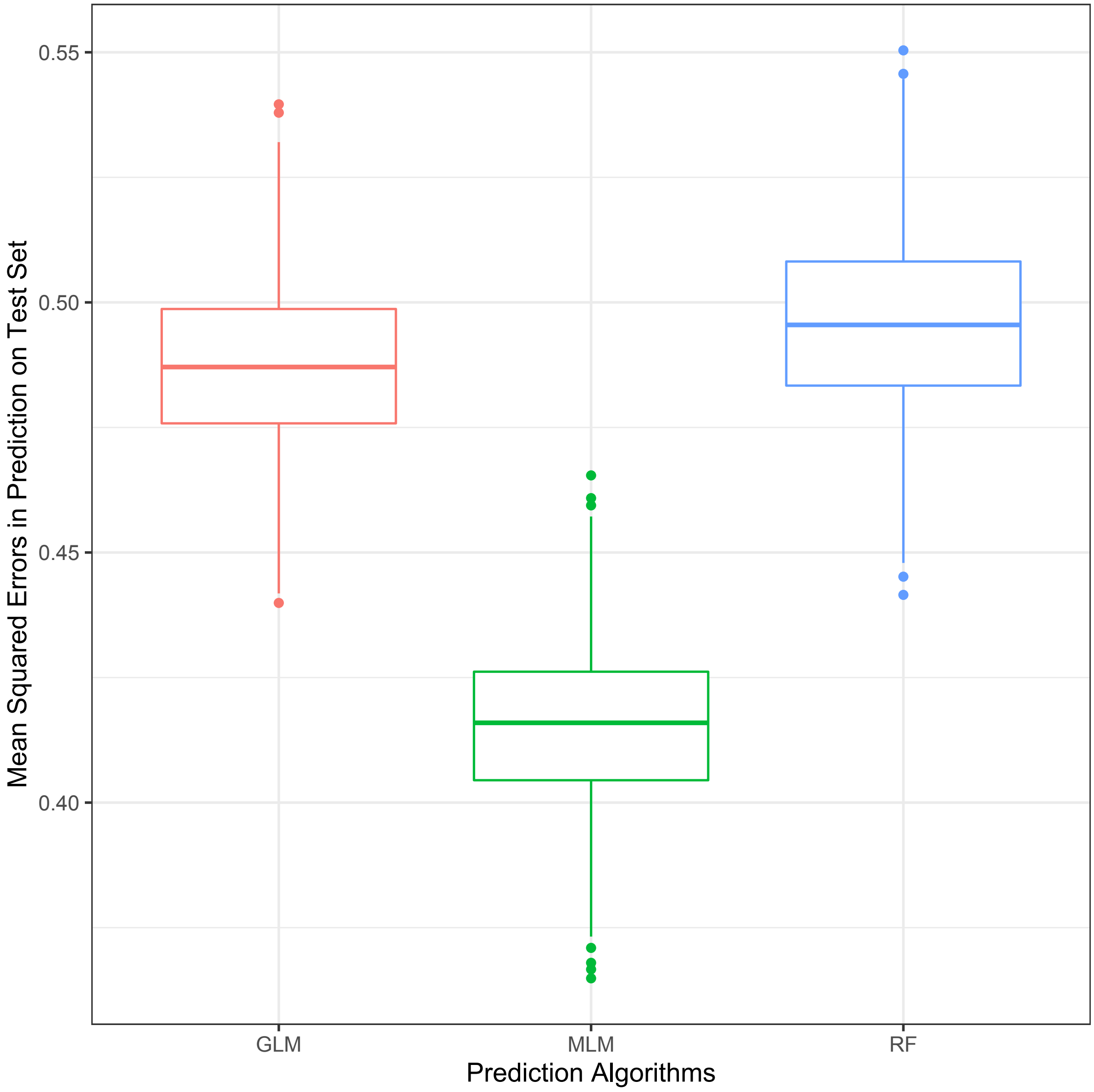

Fig. S14. Performances of three predictive algorithms for Chess in Schools. As shown in the plot, MLM has the lowest average prediction error than the other two when the target of prediction is post-test score.

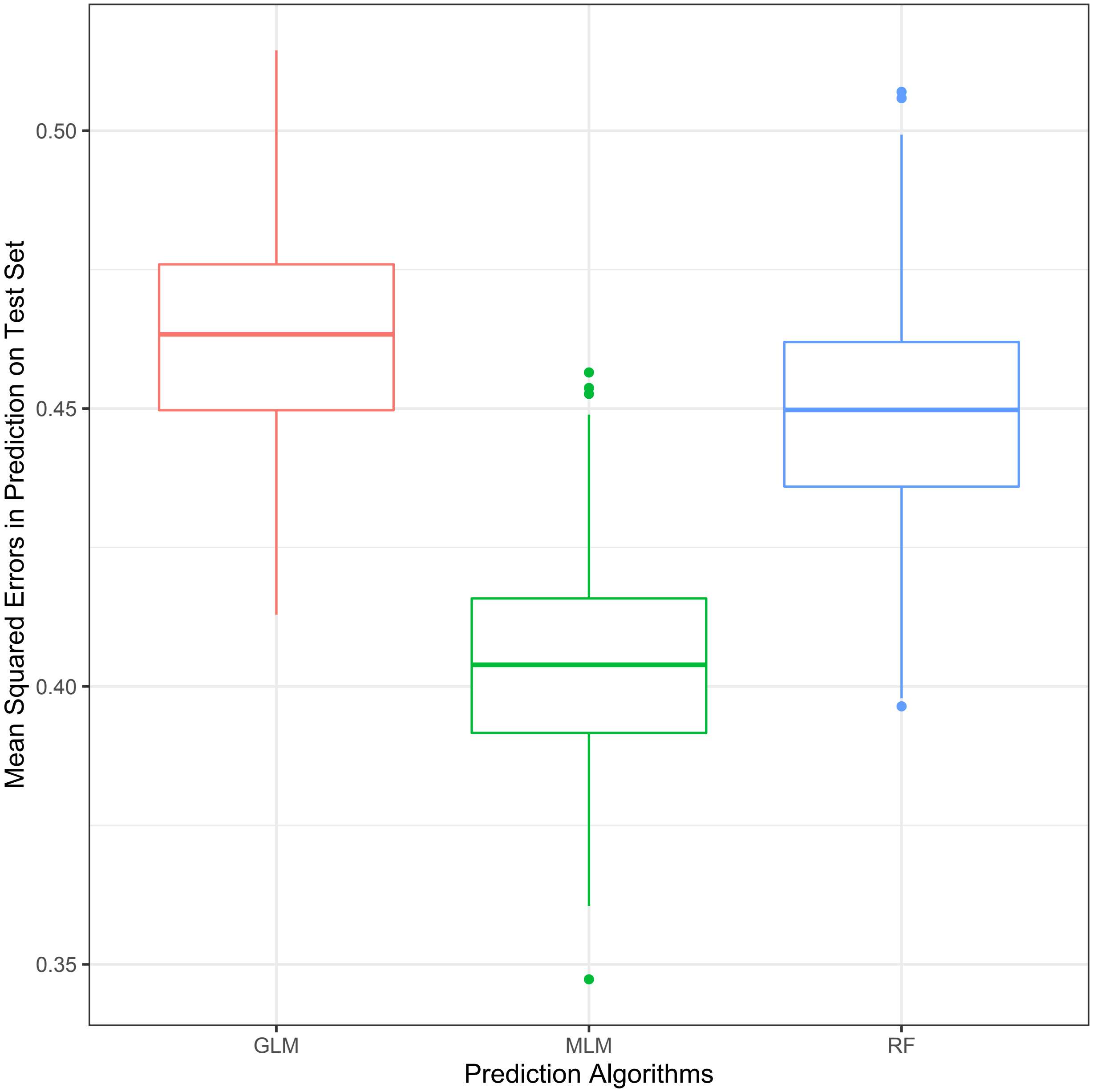

Fig. S15. Philosophy for Children. Cluster Randomised Trial, $n = 1,529$, Maths, Effect Size: 0.1, Padlock Rating: 3. Prediction errors in different datasets from different research designs. Performances of the three algorithms largely respect the research designs, but there is no guarantee that one would be always better than others on average.

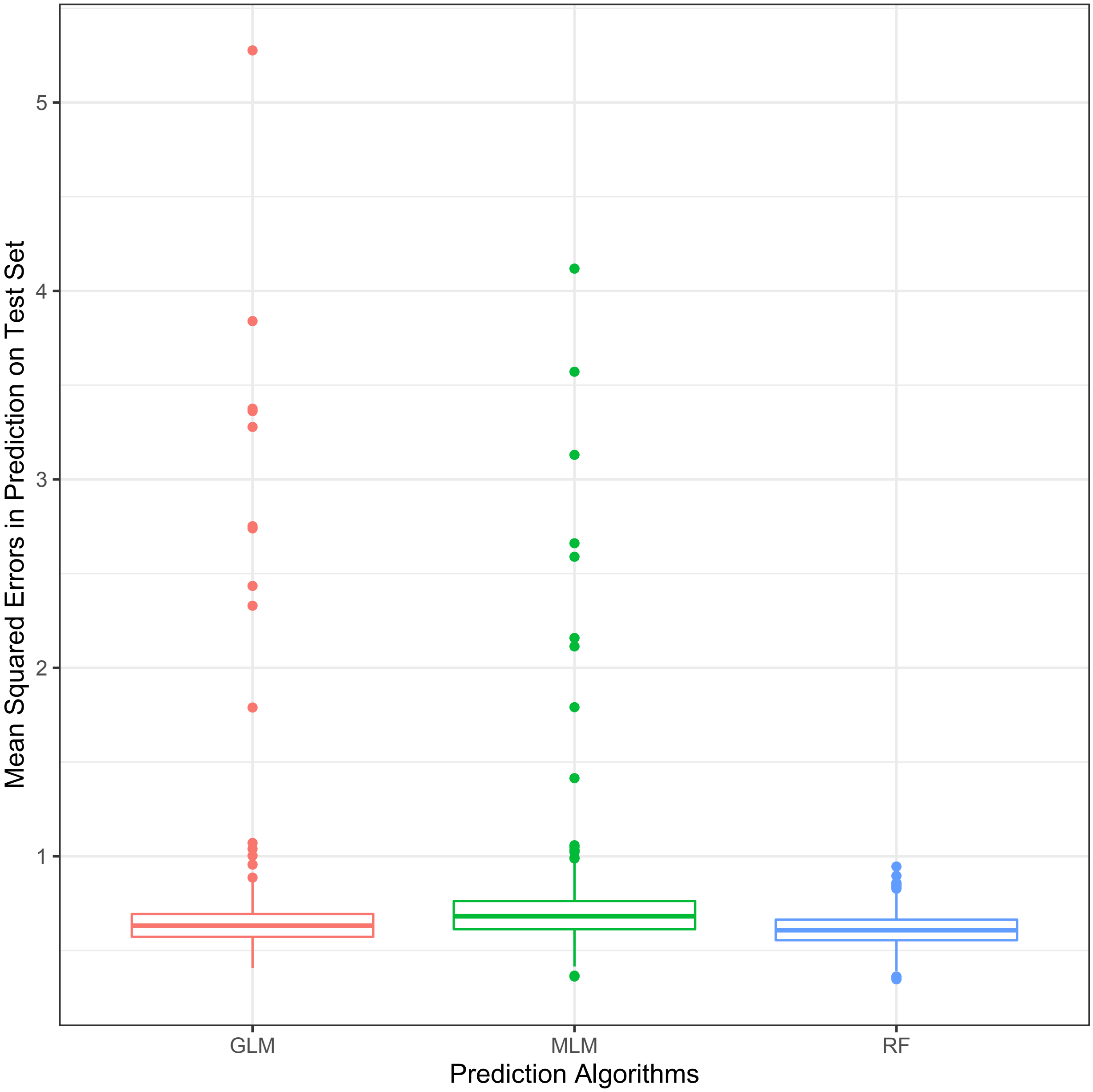

Fig. S16. Catch up Numeracy. Multi-Site Trial, $n = 216$, Maths, Effect Size: 0.21, Padlock Rating: 3. Prediction errors in different datasets from different research designs. Performances of the algorithms largely respect the research designs, but there is no guarantee that one would be always better than others on average.

Table S2. Factual and counterfactual predictions using rigorously trained algorithms. Set of parameters with the lowest average prediction error, and the other two closest to 0.5 and 1 standard deviation above the set with the lowest average prediction error. All three candidate algorithms examined.

Model Type	Performance	Min	Min + ½ sd	Min + 1 sd
**GLM**	MSE	0.44	0.45	0.46
	Which	120th	402nd	501st
	β on treatment	−0.05	−0.04	−0.06
	Average ITE (FP – CFP)	**0** (sd: 0.05)	**0** (sd: 0.04)	**0** (sd: 0.06)
	Average ITE (FO – CFP)	**0** (sd: 0.7)	**0.01** (sd: 0.7)	**0.01** (sd: 0.69)
**MLM**	MSE	0.36	0.37	0.38
	Which	854th	792nd	805th
	β on treatment	−0.07	0.01	−0.01
	Average ITE (FP – CFP)	**0** (sd: 0.07)	**0** (sd: 0.01)	**0** (sd: 0.01)
	Average ITE (FO – CFP)	**0** (sd: 0.63)	**0.01** (sd: 0.63)	**0.01** (sd: 0.62)
**RF**	MSE	0.44	0.45	0.46
	Which	414th	120th	204th
	β on treatment	NA	NA	NA
	Average ITE (FP – CFP)	**0** (sd: 0.14)	**0** (sd: 0.14)	**0** (sd: 0.13)
	Average ITE (FO – CFP)	**0.01** (sd: 0.52)	**0.01** (sd: 0.53)	**0.02** (sd: 0.53)

References

1. XL Meng, Dissecting multiple imputation from a multi-phase inference perspective: What happens when God's, imputer's and analyst's models are uncongenial? *Stat. Sinica* **27**, 1485–1594 (2017).
2. Z Xiao, A Kasim, S Higgins, Same difference? Understanding variation in the estimation of effect sizes from educational trials. *Int. J. Educ. Res.* **77**, 1–14 (2016).
3. AJ Ames, Accuracy and Precision of an Effect Size and Its Variance From a Multilevel Model for Cluster Randomized Trials: A Simulation Study. *Multivar. Behav. Res.* **48**, 592–618 (2013).
4. HS Bloom, SW Raudenbush, MJ Weiss, K Porter, Using Multisite Experiments to Study Cross-Site Variation in Treatment Effects: A Hybrid Approach With Fixed Intercepts and a Random Treatment Coefficient. *J. Res. on Educ. Eff.* **10**, 817–842 (2017).
5. JL Peugh, A practical guide to multilevel modeling. *J. Sch. Psychol.* **48**, 85–112 (2010).
6. M Petticrew, et al., Damned if you do, damned if you don't: subgroup analysis and equity. *J. Epidemiol. & Community Heal.* **66**, 95–98 (2012).
7. H Lortie-Forgues, M Inglis, Rigorous Large-Scale Educational RCTs Are Often Uninformative: Should We Be Concerned? *Educ. Res.* **48**, 158–166 (2019).
